# Combination of Betulinic Acid Fragments and Carbonic Anhydrase Inhibitors—A New Drug Targeting Approach

**DOI:** 10.3390/pharmaceutics16030401

**Published:** 2024-03-14

**Authors:** Matthias Bache, Niels V. Heise, Andreas Thiel, Anne Funtan, Franziska Seifert, Marina Petrenko, Antje Güttler, Sarah Brandt, Thomas Mueller, Dirk Vordermark, Iris Thondorf, René Csuk, Reinhard Paschke

**Affiliations:** 1 Department of Radiotherapy, Martin-Luther-University Halle-Wittenberg, D-06120 Halle (Saale), Germany; marina.petrenko@uk-halle.de (M.P.); antje.guettler@uk-halle.de (A.G.); dirk.vordermark@uk-halle.de (D.V.); 2 Institute of Chemistry, Martin-Luther-University Halle-Wittenberg, D-06120 Halle (Saale), Germany; niels.heise@chemie.uni-halle.de (N.V.H.); rene.csuk@chemie.uni-halle.de (R.C.); 3 Institute of Biochemistry and Biotechnology, Martin-Luther-University Halle-Wittenberg, D-06120 Halle (Saale), Germany; andreas.thiel@student.uni-halle.de (A.T.); iris.thondorf@biochemtech.uni-halle.de (I.T.); 4 BioCenter, Martin-Luther-University Halle-Wittenberg, D-06120 Halle (Saale), Germanyfranziska.seifert@pharmazie.uni-halle.de (F.S.); reinhard.paschke@biozentrum.uni-halle.de (R.P.); 5 Department of Internal Medicine IV (Hematology/Oncology), Martin-Luther-University Halle-Wittenberg, D-06120 Halle (Saale), Germany; sarah.brandt@uk-halle.de (S.B.); thomas.mueller@uk-halle.de (T.M.)

**Keywords:** betulin and betulinic acid, Carbonic anhydrase IX inhibition, dual tumor targeting agents

## Abstract

Human carbonic anhydrase IX (hCA IX) is a zinc(II)-dependent metalloenzyme that plays a critical role in the conversion of carbon dioxide and water to protons and bicarbonate. It is a membrane-bound protein with an extracellular catalytic center that is predominantly overexpressed in solid hypoxic tumors. Sulfamates and sulfonamides, for example acetazolamide (AZA), have been used to inhibit hCA IX in order to improve the response to solid hypoxic tumors. In the present study, we propose a new drug targeting approach by attaching the natural cytotoxic substances betulin and betulinic acid (BA) via a linker to sulfonamides. The conjugate was designed with different spacer lengths to accumulate at the target site of hCA IX. Computational and cell biological studies suggest that the length of the linker may influence hCA IX inhibition. Cytotoxicity tests of the newly synthesized bifunctional conjugates 3, 5, and 9 show effective cytotoxicity in the range of 6.4 and 30.1 µM in 2D and 3D tumor models. The hCA IX inhibition constants of this conjugates, measured using an in vitro enzyme assay with *p*-nitrophenyl acetate, were determined in a low µM-range, and all compounds reveal a significant inhibition of hypoxia-induced CA activity in a cell-based assay using the Wilbur–Anderson method. In addition, the cells respond with G1 increase and apoptosis induction. Overall, the dual strategy to produce cytotoxic tumor therapeutics that inhibit tumor-associated hCA IX was successfully implemented.

## 1. Introduction

Many pentacyclic triterpenes possess antibacterial [1], antiviral [2,3], analgesic, and antitumor activities as well as proven anti-inflammatory [4,5], anti-HIV [6], and anti-angiogenic [7,8] activities. Very interesting representatives of the lupane family are betulinic acid (BA) and betulin, which are found in the bark of birch (*Betula alba*) and plane trees (*Platanus* sp.). They have also been shown to provide antitumor activity against a wide variety of tumor cell lines, with some compounds even showing selective cytotoxicity [9,10].

Carbonic anhydrase (CA), a metalloenzyme with a zinc(II) ion in its active site, is an important enzyme in the conversion of carbon dioxide and water into bicarbonate ions and protons; it is therefore indispensable for transport, acid-base regulation, gas exchange, photosynthesis and CO_2_ fixation [11,12]. To date, eight different families of carbonic anhydrases are known (α-, β-, γ-, δ-, and ζ-carbonic anhydrase), which differ in their respective amino acid sequences, 3D tertiary structure, and the metal ion in the center. Human carbonic anhydrase IX (hCA IX), member of the α-family, plays a special role since its gene expression is up-regulated under hypoxic conditions; therefore, this enzyme is the most overexpressed carbonic anhydrase isoform in hypoxic tumors. Thus, it can be considered as a key factor in the processes of tumor development and growth. It plays a critical role in the maintenance of intracellular pH by which cancer cells adapt to the hypoxic conditions of their extracellular environment. In addition, hCA IX activity stimulates cancer cell migration and is associated with the increase in aggressive/invasive phenotypes of tumors [13].

hCA IX consists of 459 amino acids divided into a proteoglycan domain, a catalytic domain, a transmembrane region, and the intracellular C-terminus. The binding pocket of hCA IX is approximately 15 Å deep with a diameter of approximately 16 Å at the top. The zinc(II) ion in the active site is complexed with three imidazole rings of His 94, 96, and 119 and a water/hydroxide ion in a tetrahedral geometry [14]. Binding of the water/hydroxide ion or a zinc-binding group is supported by hydrogen bonding to Thr 199, which in turn interacts with Glu 106 via a hydrogen bond. These two amino acid residues are also referred to as “gatekeepers” [15,16,17].

**Figure 1:**
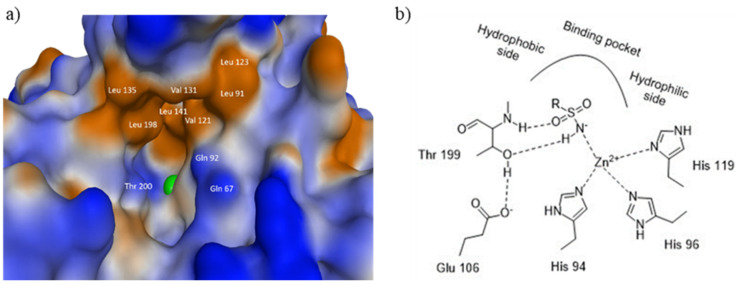
(**a**) Binding pocket of hCA IX (PDB code: 3iai) with the essential amino acids of the hydrophobic and hydrophilic site (hCA II numbering). The coloring of the surface is based on the hydrophobicity (brown: hydrophobic, grey: neutral, blue: hydrophilic). The zinc(II) ion is shown as a green sphere in the center of the binding pocket. (**b**) Sulfonamide binding mechanism of hCA IX.

As shown in Figure 1, the binding pocket of hCA IX can be divided into a hydrophobic and a hydrophilic area. The hydrophobic site of the binding pocket is formed by the amino acid residues Leu 91, Val 121, Leu 123, Val 131, Leu 135, Leu 141, and Leu 198. These residues provide the opportunity for hydrophobic interactions with an inhibitor molecule. In the opposite region of the binding pocket, in addition to the “gatekeeper” residues, the amino acid residues Thr 200, Gln 67, and Gln 92 are particularly suitable for the formation of hydrogen bonds with the inhibitor.

A variety of chemical entities have already been described in the literature as tumor-associated hCA IX inhibitors. In particular, sulfamates [18,19,20] and sulfonamides [21] are among the most important classes of hCA IX inhibitors. It has been shown that both sulfamates and sulfonamides bind to the zinc center of hCA IX at a zinc(II)-N distance of about 2 Å, while the deprotonated NH group of the inhibitor forms a hydrogen bond to the neighboring Thr 199. A second hydrogen bond is formed by the NH of Thr 199 to the oxygen of the sulfonamide/sulfamate group (Figure 1).

**Figure 2:**
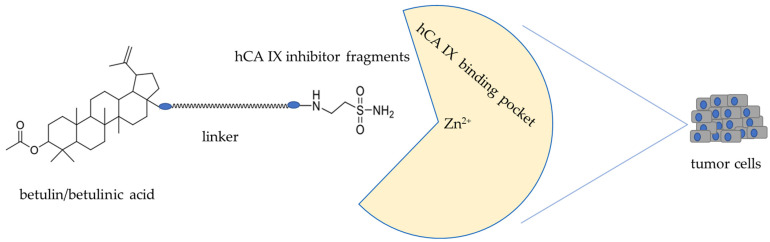
Schematic illustration of the concept of a bifunctional conjugate of betulin/BA, a linker, and CA IX inhibitor fragments.

In this work, we use the concept of small molecule drug conjugates (SMDCs) [22] for the development of potential drugs targeting hCA IX-expressing cancer cells. Krall et al. reported the first successfully tested SMDCs for the treatment of carbonic anhydrase IX-expressing tumors [23]. The present study focuses on sulfonamide inhibitors that bind directly to the zinc(II) ion in the active site of hCA IX. The linkage of this inhibitor group to the cytotoxic natural products betulin or BA via a cleavable spacer group results in bifunctional conjugates that can accumulate on the surface of tumor cells, thus leading to an enrichment of the cytotoxic agent at its site of action (Figure 2).

The type of linker will affect the overall polarity of the conjugate and hence the solubility of the compound, and it is also possible to incorporate different cleavage sites [24]. As reviewed in Yang et al., 2023 a drug conjugate consists of three core components, including a tumor-homing carrier, a spacer, and payload [24]. In our case, the tumor-homing carrier is for instance the sulfonamide group, the payload is the betulin/BA-fragment, and the spacer is the separating linker. We have chosen the spacer as the cleavable ester/amide group with a different length to find out if the ester cleavage would be influenced by the position of the ester group after the tumor-homing group has docked in the binding pocket of hCA IX. Since the prolongation of the spacer usually leads to a more lipophilic conjugate, we introduced diglycolamine to improve the solubility. So far, these are only chemical analogies and still need to be proven in practice.

To support the synthetic design studies, we performed computational studies concerning the length and flexibility of several linker molecules. The inhibitory effect of the synthesized conjugates against hCA IX was investigated determining the inhibition constant *K_i_* using an enzyme kinetic esterase assay with *p*-nitrophenyl acetate as the artificial substrate and in a cell-based system measured using the Wilbur–Anderson method. Cytotoxicity was determined in 2D and 3D tumor cell culture models. Annexin V staining and cell cycle analysis were used to assess cell death upon application of the bifunctional conjugates to the breast cancer cell line MCF-7.

## 2. Materials and Methods

### 2.1. Synthesis

#### 2.1.1. General

NMR spectra were recorded using the Varian spectrometers (Darmstadt, Germany) DD2 and VNMRS (400 and 500 MHz, respectively). MS spectra were taken on a Advion expression LCMS mass spectrometer (Ithaca, NY, USA; positive ion polarity mode, solvent: methanol, solvent flow: 0.2 mL/min, spray voltage: 5.17 kV, source voltage: 77 V, APCI corona discharge: 4.2 μA, capillary temperature: 250 °C, capillary voltage: 180 V, sheath gas: N_2_). Thin-layer chromatography was performed on pre-coated silica gel plates supplied by Macherey-Nagel (Düren, Germany). The melting points were determined using the Leica hot stage microscope Galen III (Leica Biosystems, Nussloch, Germany) and are uncorrected. The solvents were dried according to usual procedures. All dry solvents were distilled over respective drying agents, except for DMF, which was distilled and stored under argon and molecular sieve. Reactions using air- or moisture-sensitive reagents were carried out under argon atmosphere in dried glassware. Triethylamine was stored over potassium hydroxide. Betulin and BA were obtained from local vendors and used as received.

NMR spectra and ESI-MS spectra of the compounds synthesized in this work are included in the Supplementary Materials.

#### 2.1.2. General Procedure of Acetylation (GPA)

To a solution of the triterpenoic acid (1 equiv.) in dry DCM, acetic anhydride (3 equiv.), triethylamine (3 equiv.), and DMAP (cat.) were added, and the mixture was stirred at 20 °C for 1 day. The product was purified with column chromatography using petroleum ether/ethyl acetate (8.5:1.5).

#### 2.1.3. 3-O-Acetyl-betulin (**1**)

This compound was prepared as previously reported; m.p. 258–260 °C (lit.: 255–258 °C); R_F_ = 0.2 (petroleum ether/ethyl acetate; 9:1); MS (ESI, MeOH): *m*/z = 991.33 ([2M+Na]^+^) [25].

#### 2.1.4. 3-O-Acetyl-betulinic Acid (**2**)

Acetylation of BA (10.0 g, 21.8 mmol) with acetic anhydride (250 mL, 3.25 mol) for 4 h as previously described followed by column chromatography (petroleum ether/ethyl acetate, 3:1) gave **2** (8.4 g, 71%) as a colorless solid; R_F_ = 0.65 (petroleum ether/ethyl acetate; 3:1); m.p. 280–282 °C (lit.: 281–284 °C); MS (ESI, MeOH): *m/z* = 497.33 ([M-H]^−^), 995.20 ([2M-H]^−^), 1017.53 ([2M-2H+Na]^−^) [26].

#### 2.1.5. (3β)-28-{[2-(Aminosulfonyl)-ethyl]-amino}28-oxolup-20(29)-en-3-yl Acetate (**3**)

Reaction of **2** (300 mg, 0.60 mmol) with oxalyl chloride (100 µL, 148.3 mg, 1.2 mmol) as previously described followed by adding it to a solution of taurine amide (150 mg, 1.2 mmol) and triethylamine (300 µL, 215 mg, 2.0 mmol) in a mixture of dry dichloromethane (20 mL) and dry dimethylformamide (5 mL), stirring at 20 °C, usual aqu. work-up, and column chromatography (DCM/methanol, 15:1) gave **3** (247 mg, 68%) as a colorless solid R_f_ = 0.45 (hexanes/ethyl acetate; 7:3); m.p. 137–140 °C; [α]_D_ = +1.3° (*c* 0.083, MeOH); ^1^H NMR (400 MHz, CDCl_3_): δ = 6.41 (*t*, *J* = 6.1 Hz, 1H, NH), 5.19 (*s*, 2H, NH2), 4.72 (*d*, *J* = 1.5 Hz, 1H, 29-H_b_), 4.59 (*d*, *J* = 1.5 Hz, 1H, 29-H_a_), 4.50–4.42 (*m*, 1H, 3-H), 3.84–3.70 (*m*, 2H, 33-H), 3.37–3.21 (*m*, 2H, 34-H), 3.06 (*dt*, *J* = 11.3, 5.8 Hz, 1H, 19-H), 2.40 (*td*, *J* = 12.4, 3.6 Hz, 1H, 13-H), 2.03 (*s*, 3H, 32-H), 1.99–1.85 (*m*, 2H, 7-H_a_, 16-H_a_), 1.82–1.73 (*m*, 1H, 22-H_b_), 1.67 (*s*, 3H, 30-H), 1.73–1.59 (*m*, 2H, 1-H_a_, 12-H_a_), 1.61–1.52 (*m*, 3H, 2, 18-H), 1.52–1.29 (*m*, 8H, 6-H, 7-H_b_, 11-H_a_, 15-H_a_, 16-H_b_, 21-H_a_, 22-H_a_), 1.29–1.19 (*m*, 3H, 9, 11-H_b_, 21-H_b_), 1.14 (*dt*, *J* = 13.7, 3.2 Hz, 1H, 15-H_b_), 1.02–0.96 (*m*, 2H, 1-H_b_, 12-H_b_), 0.95 (*s*, 3H, 27-H), 0.92 (*s*, 3H, 26-H), 0.83 (*s*, 3H, 24-H), 0.83 (*s*, 3H, 23-H), 0.82 (*s*, 3H, 25-H), 0.80–0.74 (*m*, 1H, 5-H) ppm; ^13^C NMR (101 MHz, CDCl_3_): δ = 177.3 (C-28), 171.2 (C-31), 150.8 (C-20), 109.7 (C-29), 81.1 (C-3), 55.9 (C-17), 55.6 (C-5), 55.0 (C-34), 50.7 (C-9), 50.2 (C-18), 46.9 (C-19), 42.6 (C-14), 40.9 (C-8), 38.5 (C-1), 38.3 (C-22), 37.9 (C-10), 37.9 (C-13), 37.3 (C-4), 34.5 (C-33), 33.6 (C-7), 30.9 (C-16), 29.8 (C-21), 29.5 (C-15), 28.1 (C-23), 25.7 (C-12), 23.8 (C-2), 21.4 (C-32), 21.1 (C-11), 19.5 (C-30), 18.3 (C-6), 16.6 (C-25), 16.3 (C-26), 16.3 (C-24), 14.7 (C-27) ppm; MS (ESI, MeOH/CHCl_3_, 4:1): *m/z* = 605.0 ([M+H]^+^); analysis calcd for C_34_H_56_N_2_O_5_S (604.89): C 67.51, H 9.33, N 4.63; found: C 67.27, H 9.53, N 4.41.

#### 2.1.6. 4-{[(3beta)-3-(Acetyloxy)-lup-20(29)-en-28-yl]-oxy}-4-oxobutanoic Acid (**4**)

To a solution of **1** (1.0 g, 1.7 mmol) in dry pyridine (10 mL), a solution of succinic anhydride (0.2 g, 2.0 mmol) and DMAP (cat. amounts) in dry pyridine (5 mL) was added, and the reaction mixture was boiled under reflux for 15 h. After completion of the reaction (as checked by TLC), usual aq. work-up followed by column chromatography (DCM/methanol, 15:1) gave **4** (942 mg, 80%) as a colorless solid m.p. 122–125 °C; [α]_D_ = +12.1° (*c* 0.198, MeOH); R_F_ = 0.15 (SiO_2_, hexanes/EtOAc, 8:2); ^1^H NMR (500 MHz, CDCl_3_): δ = 4.68 (s, 1H, 29-H_a_), 4.58 (s, 1H, 29-H_b_), 4.46 (dd, J = 10.6, 5.6 Hz, 1H, 3-H_a_), 4.30 (d, J = 11.0 Hz, 1H, 28-H_a_), 3.88 (d, J = 11.1 Hz, 1H, 28-H_b_), 2.75–2.54 (m, 4H, 34-H, 35-H), 2.42 (td, J = 11.0, 5.8 Hz, 1H, 19-H), 2.03 (s, 3H, 32-H), 2.01–1.88 (m, 1H, 21-H_a_), 1.85–1.79 (m, 1H, 16-H_a_), 1.75 (dd, J = 12.5, 7.9 Hz, 1H, 22-H_a_), 1.68 (s, 3H, 30-H), 1.72–1.54 (m, 7H, 1-H_a_, 13-H, 15-H_a_, 12-H_a_, 2-H, 9-H), 1.50 (s, 1H, 6-H_a_), 1.44–1.35 (m, 5H, 6-H_b_, 11-H_a_, 21-H_b_, 7-H), 1.34–1.14 (m, 3H, 16-H_b_, 18-H, 11-H_b_), 1.02 (s, 3H, 23-H), 1.12–0.90 (m, 4H, 22-H_b_, 12-H_b_, 15-H_b_, 1-H_b_), 0.96 (s, 3H, 27-H), 0.84 (s, 3H, 24-H), 0.84 (s, 3H, 26-H), 0.83 (s, 3H, 25-H), 0.78 (m, 1H, 5-H). ppm; ^13^C NMR (126 MHz, CDCl_3_): δ = 177.8 (C-36), 172.6 (C-33), 171.3 (C-31), 150.2 (C-20), 110.0 (C-29), 81.1 (C-3), 63.3 (C-28), 55.5 (C-5), 50.4 (C-18), 48.9 (C-9), 47.9 (C-19), 46.6 (C-17), 42.8 (C-14), 41.0 (C-8), 38.5 (C-1), 37.9 (C-4), 37.7 (C-13), 37.2 (C-10), 34.2 (C-22), 29.9 (C-16), 29.1 (C-35), 28.1 (C-24), 27.2 (C-15), 25.3 (C-12), 23.8 (C-2), 21.4 (C-32), 20.9 (C-11), 19.3 (C-30), 18.3 (C-6), 16.6 (C-25), 16.2 (C-23), 14.9 (C-27) ppm; MS (ESI, MeOH): *m/z* = 583.6 (100%, [M-H]^−^); analysis calcd for C_36_H_56_O_6_ (584.84): C 77.93, H 9.65; found: C 77.71, H 9.95.

#### 2.1.7. (3β)-3-(Acetyloxy)-lup-20(29)-en-28-yl 4-{[2-(aminosulfonyl)-ethyl]amino}-4-oxobutanoate (**5**)

Compound **4** (150 mg, 0.25 mmol) was dissolved in DMF (5 mL) along with an excess of DIPEA. Under stirring at 20 °C, a stoichiometric amount of DCC dissolved in DMF (2 mL) was first added, followed by a stoichiometric amount of HOBT dissolved in DMF (3 mL). After an activation time of 10 min, taurine amide (87.5 mg, 0.7 mmol), also dissolved in DMF, was added. The reaction solution was stirred at 20 °C for an additional 15 h. Volatiles were removed under reduced pressure followed by usual aqu. work-up and chromatography (petroleum ether/ethyl acetate, 8:2) to yield **5** (162 mg, 92%) as a colorless solid; m.p. 122–126 °C; [α]_D_ = +6.7° (*c* 0.096, MeOH); R_F_ = 0.28 (SiO_2_, hexanes/EtOAc, 3:7); ^1^H NMR (500 MHz, CDCl_3_): δ = 6.69 (*s*, 1H, NH), 5.79–4.90 (*m*, 2H, NH_2_), 4.68 (*s*, 1H, 29-H_a_), 4.58 (*s*, 1H, 29-H_b_), 4.46 (*dd*, *J* = 10.4, 5.8 Hz, 1H, 3-H), 4.26 (*d*, *J* = 10.8 Hz, 1H, 28-H_a_), 3.86 (*d*, *J* = 10.7 Hz, 1H, 28-H_b_), 3.83–3.73 (*m*, 2H, 37-H), 3.42–3.24 (*m*, 2H, 38-H), 2.79–2.64 (*m*, 2H, 35-H), 2.59–2.46 (*m*, 2H, 34-H), 2.46–2.34 (*m*, 1H, 19-H), 2.03 (*s*, 3H, 32-H), 1.98–1.88 (*m*, 1H, 16-H_a_), 1.85–1.78 (*m*, 1H, 16-H_b_), 1.77–1.72 (*m*, 1H, 22-H_a_), 1.67 (*s*, 3H, 30-H), 1.70–1.54 (*m*, 7H, 1-H_a_, 2-H, 12-H_a_, 13-H, 15-H_a_, 18-H), 1.53–1.45 (*m*, 1H, 6-H_a_), 1.44–1.34 (*m*, 5H, 6-H_b_, 7-H, 11-H_a_, 21-H_a_), 1.32–1.13 (*m*, 3H, 9-H, 11-H_b_, 21-H_b_), 1.10–0.98 (*m*, 4H, 1-H_b_, 12-H_b_, 15-H_b_, 22-H_b_), 1.02 (*s*, 3H, 26-H), 0.96 (*s*, 3H, 27-H), 0.85–0.83 (*m*, 6H, 23-H, 24-H), 0.83 (*s*, 3H, 25-H), 0.78 (*d*, *J* = 9.3 Hz, 1H, 5-H) ppm; ^13^C NMR (126 MHz, CDCl_3_): δ = 173.8 (C-33), 172.8 (C-31), 171.2 (C-36), 150.1 (C-20), 110.1 (C-29), 81.1 (C-3), 63.5 (C-28), 55.5 (C-5), 54.5 (C-38), 50.4 (C-9), 48.9 (C-18), 47.8 (C-19), 46.6 (C-17), 42.8 (C-14), 41.0 (C-8), 38.5 (C-1), 37.9 (C-4), 37.7 (C-13), 37.2 (C-10), 35.1 (C-22), 34.7 (C-7), 34.3 (C-37), 31.1 (C-34), 29.9 (C-35), 29.7 (C-16), 29.6 (C-21), 28.1 (C-24), 27.2 (C-15), 25.3 (C-12), 23.8 (C-2), 21.4 (C-32), 20.9 (C-11), 19.2 (C-30), 18.3 (C-6), 16.6 (C-25), 16.3 (C-26), 16.2 (C-23), 14.9 (C-27) ppm; MS (ESI, MeOH): *m/z* = 714.0 (100%, [M+Na]^+^); analysis calcd for C_38_H_62_N_2_O_7_S (690.98): C 66.05, H 9.04, N 4.05; found: C 65.84, H 9.23, N 3.86.

#### 2.1.8. 5-Amino-1,3.4-thiadiazole-2-sulfonamide (**6**)

Acetazolamide (10.0 g, 45.2 mmol) was dissolved in conc. HCl (60 mL), and the solution was heated under reflux for 3 h. After neutralization with NaOH, saturation with NaCl, and extraction with THF removal of the organic solvent, **6** was obtained as a white solid (7.8 g, 96%); m.p. 197 °C decomp. (lit.: 195); R_F_ = 0.32 (SiO_2_, CHCl_3_/MeOH 9:1); ^1^H NMR (500 MHz, DMSO-d_6_): δ = 8.04 (s, 2H, NH2), 7.84 (s, 2H, NH2). ppm; ^13^C NMR (126 MHz, DMSO-d_6_): δ 171.7, 157.9. ppm; MS (ESI, MeOH): *m/z* = 179.0 (100%, [M-H]^−^) [27].

#### 2.1.9. 4 {[5-(Amino sulfonyl)-1,3,4-thiadiazol-2-yl]-amino}-4-oxobutanoic Acid (**7**)

To a solution of **6** (300 mg, 1.6 mmol) in acetonitrile (10 mL), succinic anhydride (170 mg, 1.6 mmol) was added. The reaction was boiled at reflux for 3.5 h and stirred for another 12 h at 20 °C. The precipitate (328 mg, 73%) was filtered off and dried; **7** was obtained as an amorphous solid; ^1^H NMR (400 MHz, DMSO-d_6_): δ = 8.28 (s, 2H, NH_2_), 2.9 (s, H, NH), 2.74 (m, 2H, 5-H), 2.58 (m, 2H, 4-H); MS (ESI, MeOH): *m/z* = 289.9 ([M+H]^+^).

#### 2.1.10. *N*-[5-(Aminosulfonyl)-1,3,4-thiadiazol-2-yl]-N’-[2-(2-hydroxyethoxy)-ethyl]-succinamide (**8**)

Following the procedure given for the synthesis of **3**, from **7** (200 mg, 0.7 mmol) and diglycolamine (70 µL, 73.6 mg, 0.7 mmol) and column chromatography (DCM/MeOH, 10:1.5), **8** (71.5 mg, 28%) was obtained as a colorless amorphous solid; R_F_ = 0.28 (DCM/MeOH, 10:1.5); ^1^H NMR (400 MHz, DMSO-d_6_): δ = 8.28 (s, 2H, NH_2_), 4.54 (s, H, OH), 3.47 (dd, 2H, 8-H), 3.38 (m, 4H, 9-H and 10-H), 3.15 (m, 4H, 4-H and 5-H); MS (ESI, MeOH): *m/z* = 368.2 ([M+H]^+^).

#### 2.1.11. (3β)-3-(Acetyloxy)-lup-20(29)-en-28-yl-4-{[5-(aminosulfonyl)-1,3,4-thiadiazol-2-yl]-amino}-4-oxobutanoate (**9**)

Compound **4** (160 mg, 0.27 mmol) was dissolved in dry DCM (10 mL), and oxalyl chloride (85 µL, 63 mg, 0.5 mmol) and catalytic amounts of DMF were added. The mixture was stirred for 2 h at 20 °C. The volatiles were removed under reduced pressure, and a solution of the residue in dry DCM (15 mL) was added to a solution of **6** (97.2 mg, 0.5 mmol) in dry DMF (2.0 mL). After stirring overnight followed by usual work-up and chromatography (DCM/MeOH, 10:0.4), **9** (100 mg, 26%) was obtained as a colorless solid; m.p. 160–164 °C; R_F_ = 0.55 (SiO_2_, hexanes/EtOAc, 7:3); ^1^H NMR (500 MHz, DMSO-d_6_): δ = 8.30 (s, 2H, NH_2_), 4.69 (s, 1H, 29-H_a_), 4.55 (s, 1H, 29-H_b_), 4.36 (dd, *J* = 11.4, 4.7 Hz, 1H, 3-H), 4.23 (d, *J* = 10.9 Hz, 1H, 28-H_a_), 3.78 (d, *J* = 11.1 Hz, 1H, 28-H_b_), 2.87–2.78 (m, 2H, 35-H), 2.76–2.65 (m, 2H, 34-H), 2.43 (s, 1H, 19-H), 1.99 (s, 3H, 32-H), 1.85 (m, 1H, 21-H_a_), 1.63 (s, 3H, 30-H), 1.76–1.43 (m, 9H, 16-H_a_, 22-H_a_, 12-H_a_, 13-H, 1-H_a_, 9-H, 15-H_a_, 2-H), 1.42–1.12 (m, 9H, 6-H, 11-H_a_, 21-H_b_, 7-H, 18-H, 16-H_b_, 11-H_b_), 0.95 (s, 3H, 23-H), 0.93 (s, 3H, 27-H), 1.09–0.73 (m, 5H, 22-H_b_, 12-H_b_, 1H_b_, 15-H_b_, 5-H), 0.79 (s, 9H, 24-H, 25-H, 26-H) ppm; ^13^C NMR (126 MHz, DMSO-d_6_): δ = 171.9 (C-31), 171.3 (C-33), 170.1 (C-36), 164.3 (C-38), 161.0 (C-37), 149.8 (C-20), 110.0 (C-29), 79.9 (C-3), 61.9 (C-28), 54.6 (C-5), 49.5 (C-18), 48.1 (C-9), 47.0 (C-19), 46.0 (C-17), 42.2 (C-14), 40.4 (C-8), 37.4 (C-4), 37.0 (C-13), 36.6 (10), 34.2, 34.0 (22), 33.5 (7), 30.9 (34), 30.0 (35), 29.1 (16), 28.9, 28.5 (21), 27.6 (24), 26.6 (C-15), (C-12), 23.4 (C-2), 21.0 (C-32), 20.3 (C-11), 18.7 (C-30), 17.7 (C-6), 16.4 (C-25), 15.8 (C-26), 15.5 (C-23), 14.5 (C-27) ppm; MS (ESI, MeOH): *m/z* = 745.7 (100%, [M-H]^−^); analysis calcd for C_38_H_58_N_4_O_7_S_2_ (747.02): C 61.10, H 7.83, N 7.50; found: C 60.87, H 7.99, N 7.36.

#### 2.1.12. (3β)-28-[2-(2-Hydroxyethoxy)-ethoxy]-lup-20(29)-en-3-yl Acetate (**10**)

From the reaction of **2** (1.0 g 2 mmol) in dry DCM (20 mL) with oxalyl chloride (470 µL, 317.3 mg, 2.5 mmol) as described above followed by the addition of diglycolamine (278 µL, 262.8 mg, 2.5 mmol) and triethylamine (184 µL, 252.5 mg, 2.5 mmol) in dry DCM (10 mL), usual aq. work-up, and chromatography (DCM/MeOH, 100:1), **10** (980 mg, 84%) was obtained as a colorless solid; R_F_ = 0.38 (DCM/MeOH, 10:0.1); m.p. 205 °C; [α]_D_ = +13.0° (*c* 0.170, MeOH); ^1^H NMR (400 MHz, CDCl_3_): δ = 6.02 (*t*, *J* = 5.2 Hz, 1H, NH), 4.72 (*d*, *J* = 1.4 Hz, 1H, 29-H_b_), 4.58 (*d*, *J* = 1.4 Hz, 1H, 29-H_a_), 4.49–4.41 (*m*, 1H, 3-H), 3.73 (*s*, 2H, 36-H), 3.60–3.52 (*m*, 4H, 34-H, 35-H), 3.53–3.44 (*m*, 1H, 33-H_b_), 3.44–3.34 (*m*, 1H, 33-H_a_), 3.10 (*td*, *J* = 11.0, 3.9 Hz, 1H, 19-H), 2.42 (*td*, *J* = 12.8, 3.4 Hz, 1H, 13-H), 2.21 (*s*, 1H, OH), 2.02 (*s*, 3H), 2.00–1.86 (*m*, 2H, 16-H_a_, 21-H_b_), 1.78–1.67 (*m*, 3H, 1-H_b_, 12-H_b_, 22-H_b_), 1.67 (*s*, 3H, 30-H), 1.64–1.52 (*m*, 4H, *2-H*, 18-H, 21-H_a_), 1.51–1.44 (*m*, 2H, 6-H_a_, 15-H_a_), 1.44–1.28 (*m*, 6H, 6-H_b_, *7-H*, 11-H_a_, 16-H_b_, 22-H_a_), 1.26 (*s*, 1H, 9-H), 1.22 (*td*, *J* = 12.2, 4.0 Hz, 1H, 11-H_b_), 1.16–1.09 (*m*, 1H, 15-H_b_), 1.05–0.97 (*m*, 2H, 1-H_a_, 12-H_a_), 0.95 (*s*, 3H, 27-H), 0.92 (*s*, 3H, 26-H), 0.84–0.82 (*m*, 6H, 23, 24-H), 0.81 (*s*, 3H, 25-H), 0.79–0.75 (*m*, 1H, 5-H) ppm; ^13^C NMR (101 MHz, CDCl_3_): δ = 176.5 (C-28), 171.1 (C-31), 151.0 (C-20), 109.5 (C-29), 81.1 (C-3), 72.3 (C-35), 70.3 (C-34), 61.9 (C-36), 55.9 (C-17), 55.6 (C-5), 50.7 (C-9), 50.2 (C-18), 47.0 (C-19), 42.6 (C-14), 40.9 (C-8), 39.1 (C-33), 38.5 (C-1), 38.5 (C-22), 37.9 (C-4), 37.9 (C-13), 37.3 (C-10), 34.5 (C-7), 33.8 (C-21), 31.0 (C-16), 29.5 (C-15), 28.1 (C-23), 25.7 (C-12), 23.8 (C-2), 21.4 (C-32), 21.1 (C-11), 19.6 (C-30), 18.3 (C-6), 16.6 (C-25), 16.3 (C-24, 26), 14.7 (C-2) ppm; MS (ESI, MeOH): *m/z* = 584.5 ([M-H]^−^); 620.5 ([M+Cl]^−^); analysis calcd for C_36_H_59_NO_5_ (585.87): C 73.80, H 10.15, N 2.39; found: C 73.62, H 10.30, N 2.11

#### 2.1.13. 4-[2-2(2-{[(3β)-3-(Acetyloxy]-lup-20(29)-en-28-yl]-oxy}ethoxy)ethoxy]4-oxobutanoic Acid (**11**)

From the reaction of **10** (500 mg, 0.85 mmol) with succinic anhydride (430 mg, 4.3 mmol) as described above followed by usual aq. work-up and chromatography (DCM/MeOH, 10:0.5), **11** (437 mg, 75%) was obtained as a colorless solid; R_F_ = 0.48 (DCM/MeOH, 10:0.5); m.p. 78–82 °C; [α]_D_ = +16.1° (*c* 0.118, MeOH); ^1^H NMR (400 MHz, CDCl_3_): δ = 6.06 (*t*, *J* = 5.5 Hz, 1H, NH), 4.72 (*d*, *J* = 1.6 Hz, 1H, 29-H_b_), 4.58 (*d*, *J* = 1.4 Hz, 1H, 29-H_a_), 4.49–4.43 (*m*, 1H, 3-H), 4.26–4.23 (*m*, 2H, 36-H), 3.66–3.62 (*m*, 2H, 35-H), 3.55–3.50 (*m*, 2H, 34-H), 3.49–3.44 (*m*, 1H, 33-H_a_), 3.43–3.37 (*m*, 1H, 33-H_b_), 3.09 (*td*, *J* = 11.0, 4.0 Hz, 1H, 19-H), 2.70–2.62 (*m*, *J* = 3.8 Hz, 4H, 38-H, 39-H), 2.46–2.38 (*m*, 1H, 13-H), 2.03 (*s*, 3H, 32-H), 2.00–1.86 (*m*, 2H, 16-H_a_, 21-H_b_), 1.78–1.69 (*m*, 2H, 1-H_b_, 12-H_a_), 1.67 (*s*, 3H, 30-H), 1.65–1.52 (*m*, 4H, 2-H, 18-H, 22-H_a_), 1.52–1.44 (*m*, 3H, 6-H_a_, 15-H_a_, 21-H_a_), 1.44–1.29 (*m*, 6H, 6-H_b_, *7-H*, 11-H_a_, 16-H_b_, 22-H_b_), 1.26 (*d*, *J* = 7.7 Hz, 2H, 9, 11-H_b_), 1.16–1.10 (*m*, 1H, 15-H_b_), 1.00 (*s*, 2H, 1-H_a_, 12-H_b_), 0.95 (*s*, 3H, 27-H), 0.92 (*s*, 3H, 26-H), 0.83 (*s*, 3H, 25-H), 0.83 (*s*, 3H, 23-H), 0.82 (*s*, 3H, 24-H), 0.79–0.76 (*m*, 1H, 5-H) ppm; ^13^C NMR (101 MHz, CDCl_3_): δ = 176.8 (C-28), 176.4 (C-40), 172.2 (C-37), 171.2 (C-31), 151.0 (C-20), 109.6 (C-29), 81.1 (C-3), 70.1 (C-34), 69.0 (C-35), 63.9 (C-36), 55.9 (C-17), 55.6 (C-5), 50.7 (C-9), 50.2 (C-18), 46.9 (C-19), 42.6 (C-14), 40.9 (C-8), 39.1 (C-33), 38.5 (C-1), 38.5 (C-22), 37.9 (C-4), 37.9 (C-13), 37.3 (C-10), 34.5 (C-7), 33.8 (C-21), 31.0 (C-16), 29.5 (C-15), 29.1 (C-39), 29.0 (C-38), 28.1 (C-23), 25.7 (C-12), 23.8 (C-2), 21.4 (C-32), 21.1 (C-11), 19.6 (C-30), 18.3 (C-6), 16.6 (C-25), 16.3 (C-26), 16.3 (C-24), 14.7 (C-27) ppm; MS (ESI, MeOH): *m/z* = 684.6 ([M-H]^−^); analysis calcd for C_40_H_63_NO_8_ (685.94): C 70.04, H 9.26, N 2.04; found: C 69.85, H 9.35, N 1.79.

#### 2.1.14. 2-(2-{[(3β)-3-Acetyloxy)-lup-20(29)-en-28-yl]oxy}ethoxy)ethyl 4-{[5-(amino-sulfonyl)-1,3,4-thiadiazol-2-yl}4-oxobutanoate (**12**)

Reaction of **11** (250 mg; 0.36 mmol) with oxalyl chloride (50 µL, 74 mg, 0.6 mmol) and **5** (97.2 mg, 0.5 mmol) followed by usual aq. work-up and chromatography (DCM/MeOH, 20:1) gave 12 (225 mg, 74%) as a colorless solid (R_F_ = 0.10 (CHCl_3_/MeOH, 95:5); m.p. 92–94 °C; [α]_D_ = +9.5° (c 0.082, MeOH); ^1^H NMR (500 MHz, CDCl_3_): δ = 12.19 (*s*, 1H, NH_b_), 6.75 (*s*, 2H, NH2), 6.19 (*t*, *J* = 5.5 Hz, 1H, NH_a_), 4.71 (*s*, 1H, 29-H_b_), 4.58 (*s*, 1H, 29-H_a_), 4.46 (*dd*, *J* = 10.3, 5.8 Hz, 1H, 3-H), 4.25 (*dd*, *J* = 3.3 Hz, 2H, 36-H), 3.67 (*t*, *J* = 4.3 Hz, 2H, 35-H), 3.55 (*t*, *J* = 5.1 Hz, 2H, 34-H), 3.42 (*qq*, *J* = 13.8, 5.2 Hz, 2H, 33-H), 3.06 (*td*, *J* = 10.9, 4.3 Hz, 1H, 19-H), 2.97 (*t*, *J* = 6.0 Hz, 2H, 38-H), 2.81 (*t*, *J* = 6.4 Hz, 2H, 39-H), 2.40 (*td*, *J* = 12.6, 3.2 Hz, 1H, 13-H), 2.03 (*s*, 3H, 32), 1.99 (*dt*, *J* = 12.3, 2.5 Hz, 1H, 21-H_b_), 1.94–1.88 (*m*, 1H, 16-H_a_), 1.80–1.73 (*m*, 1H, 1-H_b_), 1.71–1.62 (*m*, 2H, 12-H_b_, 22-H_b_), 1.67 (*s*, 3H, 30-H), 1.63–1.52 (*m*, 4H, *2-H*, 18, 21-H_a_), 1.52–1.28 (*m*, 8H, *6*-H, *7*-H, 11-H_a_, 15-H_b_, 16-H_b_, 22-H_a_), 1.28–1.23 (*m*, 1H, 9-H), 1.21 (*dt*, *J* = 12.4, 3.9 Hz, 1H, 11-H_b_), 1.13 (*d*, *J* = 13.2 Hz, 1H, 15-H_a_), 1.04–0.96 (*m*, 2H, 1-H_a_, 12-H_a_), 0.95 (*s*, 3H, 27-H), 0.91 (*s*, 3H, 26-H), 0.84–0.81 (*m*, 6H, 23, 24-H), 0.81 (*s*, 3H, 25-H), 0.77 (*d*, *J* = 9.7 Hz, 1H, 5-H) ppm; ^13^C NMR (126 MHz, CDCl_3_): δ = 177.1 (C-28), 172.7 (C-31), 171.2, 170.9 (C-40), 164.6 (C-42), 162.7 (C-41), 150.9 (C-20), 109.7 (C-29), 81.1 (C-3), 70.2 (C-34), 68.9 (C-35), 64.3 (C-36), 55.9 (C-17), 55.6 (C-5), 50.6 (C-9), 50.2 (C-18), 47.0 (C-19), 42.6 (C-8), 40.9 (C-14), 39.2 (C-33), 38.5 (C-1, C-22), 38.0 (C-13), 37.9 (C-4), 37.3 (C-10), 34.5 (C-7), 33.8 (C-21), 31.0 (C-38), 30.9 (C-16), 29.5 (C-15), 28.9 (C-39), 28.1 (C-23), 25.7 (C-12), 23.8 (C-2), 21.5 (C-32), 21.1 (C-11), 19.5 (C-30), 18.3 (C-6), 16.6 (C-25), 16.3 (C-26), 16.3 (C-24), 14.8 (C-27)) ppm; MS (ESI, MeOH/CHCl_3_ (4:1)): *m/z* = 846.9 (100%, [M-H]^−^); analysis calcd for C_42_H_65_N_5_O_9_S_2_ (843.13): C 59.48, H 7.73, N 8.26; found: C 59.20, H 7.90, N 8.03.

### 2.2. Cell Lines and Treatment with Betulin and BA Derivatives

Human cancer cell lines and normal fibroblast cell line NIH 3T3 (mouse) were cultured in RPMI 1640 medium containing L-glutamine (Capricorn Scientific, Ebsdorfergrund, Germany), 10% FBS (Sigma-Aldrich, St. Louis, MO, USA), and 1× penicillin and streptomycin (Capricorn Scientific) at 37 °C and 5% CO_2_. All of the compounds were dissolved in dimethyl sulfoxide (DMSO) to achieve a 20 mM stock solution. Cells were seeded in cell culture flasks 24 h before treatment. The treatment was performed for 24 h. 

### 2.3. Cytotoxicity

The cytotoxicity of the drugs was determined in 2D tumor models using sulforhodamine B (SRB) assays. Treatment started 24 h after seeding the tumor cells in 96-well plates as previously described [28]. 

To determine cytotoxicity in the 3D tumor models, spheroid cytotoxicity assays were performed as previously described [29]. The luciferase-expressing, breast cancer cell lines MDA-MB-231 and Hs578T and derived spheroids were cultivated in RPMI medium (Sigma-Aldrich, Taufkirchen, Germany) containing 10% fetal bovine serum (BioWest, Nuaillé, France) and 10% penicillin/streptomycin (Sigma-Aldrich) at 37 °C/5% CO_2_ in a humid atmosphere. For generation of single tumor spheroids, tumor cells resuspended in culture medium were seeded into 96-well plates, which were coated before with 0.7% agarose (SeaKem^®^ GTG™ Agarose, Lonza, Basel, Switzerland). After the formation of spheroids (48 h), they were treated with serial dilutions of compounds. Measurements of luciferase activity were performed on day 7, after supplementation with D-luciferin (Perkin Elmer, Rodgau, Germany), on a Tecan Spark microplate reader (Tecan, Männedorf, Switzerland). Generation of dose-response curves and calculation of IC_50_ values including standard deviations were accomplished using GraphPad Prism8.

### 2.4. Annexin V Assay

The Annexin V assays were performed as previously described [28].

### 2.5. Cell Cycle

Cell cycle analyses were performed as previously described [28].

### 2.6. Human Carbonic Anhydrase IX (hCAIX) Inhibition

In this measurement, the enzymatically catalyzed hydrolysis of *p*-nitrophenyl acetate to *p*-nitrophenol was investigated. Subsequent pH-dependent dissociation to *p*-nitrophenolate results in a color change from colorless to yellow with an absorption maximum at λ = 405 nm. In the esterase assay, the change in absorbance at λ_max_ = 405 nm is measured as a function of time and substrate concentration. In the present study, we used recombinant human carbonic anhydrase IX (SinoBiological; Cat.: 10107-H08H) with a specific activity of 79 nmol/min mg^−1^. Activity measurements were performed in 12.5 mM Tris buffer at pH 7.5, a constant temperature of T = 20 °C, and a wavelength of 405 nm. In the first step, the progress curves for the v-S-characteristic were measured without inhibitor. For this, 149.8 µL of Tris buffer (pH = 7.5), 6.8 µL of ACN, and 10 µL of enzyme (5.1 µM) were added to one well each of a 96-half area-well plate, incubated for three minutes at 20 °C, and then 3.4 µL of the appropriate substrate solution (25 mM–125 mM) was added using an 8-channel pipette to start the reaction. This was followed by measurement of the v-S-characteristics with inhibitor. For this, 149.8 µL of Tris buffer together with 3.4 µL of ACN, 3.4 µL of the inhibitor of interest (1.25 M–200 µM), and 10 µL of enzyme (5.1 µM) were added to one well each of a 96-half area well plate and incubated for three minutes at 20 °C. Then, as in the previous two steps, 3.4 µL of the appropriate substrate solution (25 mM–125 mM) was added using an 8-channel pipette to start the reaction. For each run, 2 experimental series (biological replicate) were prepared simultaneously and measured for 10 min at a measurement interval of 8 s, also as a two-fold determination (technical replicate). For all steps, the change of absorbance at 405 nm over time was measured in a TECAN plate reader. The resulting reaction velocities, calculated from a calibration curve with *p*-nitrophenole under the same conditions, were plotted against the substrate concentration. The kinetic evaluation for competitive inhibition was then performed with the program GraphPad Prism.

### 2.7. Cell-Based Measurement of hCA IX Inhibition

In a cell-based system, the changes of extracellular pH due to activity of hypoxia-induced hCA IX were determined. Hydration of CO_2_ to hydrogen carbonate and a proton catalyzed by extracellular hCA IX are the basis for detecting changes in pH depending on activity of hCA IX. Breast cancer cells (Hs578T) were cultured under hypoxic conditions for induction of hCA IX expression and afterwards treated with 20 µM of each substance for 3 h. Normoxic and hypoxic cells without treatment were used as reference. Cells were washed, scraped down, and resuspended in ice-cold isotonic buffer. One minute after extracellular pH measurements started in stirred cells, CO_2_-saturated water was added, and the duration (T) to lower the pH of the isotonic buffer from 8.0 to 6.6 at 4 °C was determined. hCA IX activity was calculated according to the Wilbur–Anderson method (WAU/mg = 2_(T_0_ – T)/T × mg protein) [T_0_, unanalyzed reaction (isotonic buffer); T, catalyzed reaction (e.g., normoxia, hypoxia, treatment)].

### 2.8. Computational Studies

The structure of derivatives **3**, **5**, **9**, and **12** were generated manually in MOE by modifying the crystal structure (CSD: HEHPAN) of BA.

Docking was done in MOE (The Molecular Operating Environment) Version 2022.02, Chemical Computing Group Inc. (Montreal, QC, Canada)] using a crystal structure of human carbonic anhydrase IX (PDB ID: 3iai), which was first subjected to the structure preparation tool of MOE to delete unbound water molecules and to add hydrogen atoms. The docking process used the sulfonamide group as the pharmacophore and the boundaries of the binding pocket to define the excluded volume. The compounds were docked in a deprotonated state. The selection of the poses was based on the scoring values and visual observation.

The structures of **9** and **12** were parameterized with Antechamber from the AMBER suite. For the MD simulations, the general AMBER force field (GAFF) [30] was used. The system was solvated in an octahedral box with TIP3P water molecules with an extension of 16 Å from the conjugate. Na^+^ and Cl^−^ ions were added for a salt concentration of 150 mM. The protocol for the simulation included minimization steps, relaxation steps, and a production run of 400 ns. Four independent simulations were performed for each conjugate. The minimization and relaxation of the system included the following steps: (1) minimization using 20,000 cycles and 10 kcal mol^−1^ Å^−2^ constraints on everything but water, (2) heating the system for 100 ps from 200 K to 310 K (solute force constant 10 kcal mol^−1^ Å^−2^) to move the water molecules, (3) system-wide minimization with 20,000 cycles, (4) heating the system from 0 K to 310 K in 0.5 ns (200,000 steps heating + 50,000 steps relaxation) with 5 kcal mol^−1^ Å^−2^ restraints on the solute, NVT, and Langevin thermostat, (5–7) stepwise reduction of solute restraints from 2.5 kcal to 1 kcal to 0 kcal mol^−1^ Å^−2^ with 0.5 ns simulation time for each step, NVT and Langevin thermostat (8) 5 ns relaxation at 310 K and 1 atm (NPT) with Langevin thermostat. The production run was under NPT conditions (1 atm) at a temperature of 310 K. For the MD simulation a, step size of 2 fs was chosen, and the trajectories were saved every 10 ps. The SHAKE algorithm was used to constrain bonds involving hydrogen. The processing and analyzing of the MD simulations was done with cpptraj [31]. 

## 3. Results and Discussion

### 3.1. Chemistry

#### 3.1.1. Synthesis of Conjugates

**Scheme 1:**
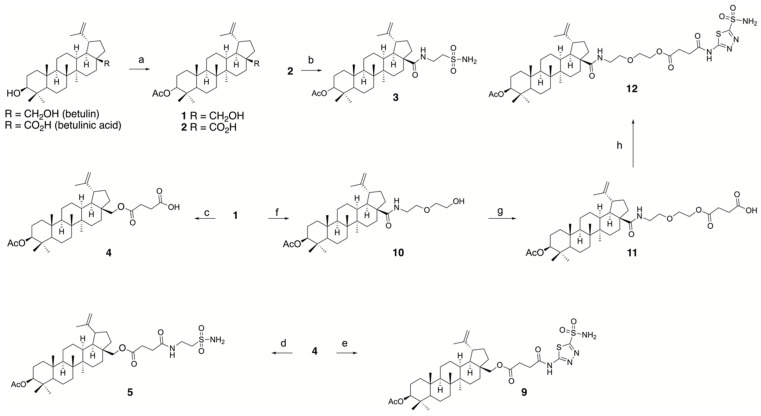
Synthesis of the sulfonamides of betulin (**5**) and BA (**3**) and the triterpenoid-acetazolamide conjugates (**9** and **12**). (a) Dry DCM, acetic anhydride, triethylamine (3 equiv.), DMAP (catal.), 20 °C for 24 h; (b) dry DCM, oxalyl chloride, taurine amide, triethylamine, overnight, 20 °C; (c) pyridine, DMAP, succinic anhydride, 5 h reflux; (d) DMF, HOBT, DCC, taurine amide, 15 h, 20 °C; (e) (i) DCM, oxalyl chloride, 2 h, (ii) DMF, DMAP (cat.) **6**; (f) (i) DCM, oxalyl chloride, 20 min (ii) 0 °C, diglycol amine; (g) pyridine, DMAP, succinic anhydride, 5 h reflux; (h) (i) DCM, oxalyl chloride, 2 h, (ii) DMF, DMAP (cat.) **6**.

According to our aim—finding new bifunctional conjugates with an antitumor and a hCA IX-inhibiting fragment using a spacer component to connect the two functional entities—we chose betulin and BA as the cytotoxic part and 2-aminoethane-1-sulfonamide and 5-amino-1,3,4-thiadiazole-2-sulfonamide as the inhibiting part. Scheme 1 describes the synthesis of the conjugates; different spacer lengths were used to test whether this change has an influence on cytotoxicity or CA inhibition.

#### 3.1.2. Synthesis of the Inhibitory Fragments of the Conjugate

**Scheme 2:**
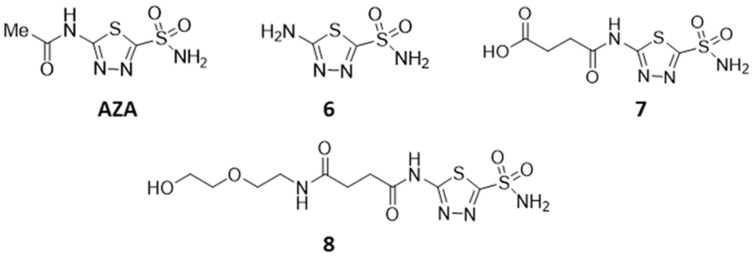
AZA and inhibitory fragments **6**–**8**.

In order to find out whether the CA-inhibiting parts of the molecule retain their inhibitory activity after the conjugates are cleaved and thus are able to block hCA IX even after the separation of the cytotoxic fragment, we synthesized some of the inhibitor fragments (Scheme 2). Being aware that compound **8** is not fully comparable to the cleaved part of compound **12**, but in terms of estimating the ability of this fragment to block carbonic anhydrase, it seemed to be useful. In this context, it was necessary to determine the cytotoxic properties of these partial fragments. Therefore, all compounds were also evaluated for their cytotoxic properties using SRB assays with an incubation time of 96 h (Table 1).

### 3.2. Cytotoxic Effects of Conjugates and Fragments

SRB assays showed the cytotoxicity of similar betulin sulfamates to be in the same order of magnitude as that of parent BA [18,32]. Slightly enhanced cytotoxicity was determined for the sulfonamides of betulin and BA [21]. Corresponding studies with the new sulfonamides **3** and **5** showed that they have similar IC_50_ values between 6.4 μM and 11.4 μM, respectively (Table 1). Table 1 also reveals that the attachment of a spacer (compounds **4**, **10**, **11**) did not significantly alter the IC_50_ values, and the addition of a taurine amide or a 5-amino-1,3.4-thiadiazole-2-sulfonamide fragment did not lead to changes in cytotoxicity (compounds **5**, **9**). An exception to these observations was found for compound **12**; for this compound, its IC_50_ value was significantly diminished. In addition, new sulfonamide **3** showed increased tumor selectivity. 

The selectivity index (SI) of the hit compounds (**3, 5** and **9**) against tumor cell lines was determined by the ratio of the IC_50_ value of NIH3 fibroblast cells to the IC_50_ values of the tumor cell lines. The SI of compound **3** for the investigated tumor cell lines was 1.4 to 2.2, whereas the tumor selectivity of compounds **5** and **9** was lower (selectivity index is between 0.9 and 1.3).

In addition, selected compounds were tested in 3D spheroid models to prove potential antitumor activity and the ability to overcome resistance to conventional chemotherapeutic drugs. Tumor spheroids are useful in vitro models representing several important aspects of real tumor tissues, i.e., three-dimensional growth with structural organization and physiologically relevant cell–cell and cell–matrix interactions; establishment of tumor microenvironmental characteristics, such as nutrient gradients, hypoxia and acidosis; as well as elucidation of drug resistance mechanisms [33,34]. For this purpose, we used our model comprising two breast cancer cell line derived spheroid types, MDA-MB-231 and Hs578T, with the latter one showing 60-fold and 20-fold resistance to the clinically relevant drugs doxorubicin and paclitaxel, respectively [29]. Since the tumor cells stably express luciferase, analysis of spheroid growth and response to therapy is accomplished by measurement of bioluminescent signal intensity. The results are compiled in Table 2. Compound **5** and **9**, representing a betulin sulfonamide and a betulin acylazolamide with similar spacer lengths, showed comparable cytotoxicity, which was in accordance with the results obtained in the 2D models. Thus, they outperformed the established CA IX/CA XII specific inhibitor U-104, which was tested in phase I/II clinical trials in patients with advanced solid tumors [35]. Interestingly, both compounds were able to reduce the high resistance of Hs578T spheroids versus MDA-MB-231 spheroids to conventional chemotherapeutic drugs to only about three-fold differences. In addition, the inhibitor fragments **7** and **8** as well as the long linker containing compound **12** exerted no cytotoxicity in the spheroid model, in accordance with the 2D model.

### 3.3. Annexin V Staining and Cell Cycle Analysis

**Figure 3:**
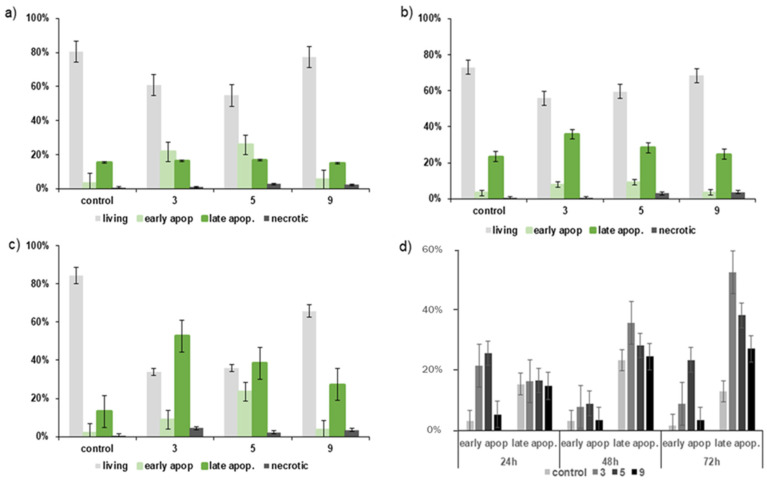
Graphical representation of Annexin V analysis of the breast cancer cell line MCF-7 after (**a**) 24 h, (**b**) 48 h, and (**c**) 72 h of treatment with compounds **3**, **5**, and **9** and an untreated control. (**d**) Summary of early and late apoptotic cells of all three time points. Double the IC_50_ concentration was used for treatment.

Compounds **3**, **5**, and **9** were used to study cell death mechanisms and cell cycle analysis. A feature that can be used as it is only characteristic of apoptotic but not necrotic cells is Annexin V staining. During apoptosis, in addition to cell shrinkage, chromatin condensation [36], and the formation of apoptotic bodies [37], phosphatidylserine, which is originally inside the membrane, is translocated to the outside. There is a rapid change in phospholipid symmetry [38] in the cell membrane, a process occurring so rapidly that the barrier function of the cell membrane is initially still present [39]. The cellular protein Annexin V can bind to the displaced phosphatidylserine in the presence of calcium [40]. To test for the presence of the cell membrane barrier, the non-membrane-permeable dye propidium iodide was added, which intercalates into the DNA. The flow cytometric data were divided into four areas (live cells, early and late apoptotic cells, and necrotic cells). Untreated cells were used as controls. The results are depicted in Figure 3. After a treatment period of 24 h, compounds **3** and **5** showed a similar effect on cell survival with 55–60% live cells, 20–25% early apoptotic cells, and 16% late apoptotic cells. In contrast, treatment with compound **9** showed no difference after 24 h compared to the untreated control. Even after 48 h, no significant difference between compound **3** and **5** was detected (55–60% live cells, 8% early apoptotic cells, 28–36% late apoptotic cells, 0.5–3% necrotic cells). After 72 h of treatment, the strongest reaction was observed with compound **3** (34% live cells, 9% early apoptotic cells, 53% late apoptotic cells, 4.5% necrotic cells). Treatment with compound **5** also showed a comparably strong effect after 72 h. Since both compounds are sulfonamides of betulin, the similar results are not surprising. This experiment also revealed that the length of the linker exerted no significant effect on the cell death mechanism. Treatment with compound **9** resulted in a comparatively smaller decrease in cell viability after 72 h. These results confirm previous studies showing that the more polar the substituent is, the later apoptosis was initiated [41].

Changes in the genetic material and the resulting mutations, which can affect the formation of tumors, usually occur during the proliferation process in the cell. A human cell passes through all four phases of this cell cycle within 20 h, with the set of its chromosomes varying between single and double. During apoptosis, fragmentation of DNA occurs along with an associated decrease in chromosome content. Fixation of cells with ethanol leads to permeabilization of the cell membrane and thus to loss of smaller DNA fragments, so that apoptotic cells contain less than the single set of chromosomes detected by a SubG1 peak. Given the different amounts of DNA in the cells and the proportional binding of propidium iodide to the DNA, the different fluorescence intensities can be used to infer the distribution of cells in the respective phases of the cell cycle.

**Figure 4:**
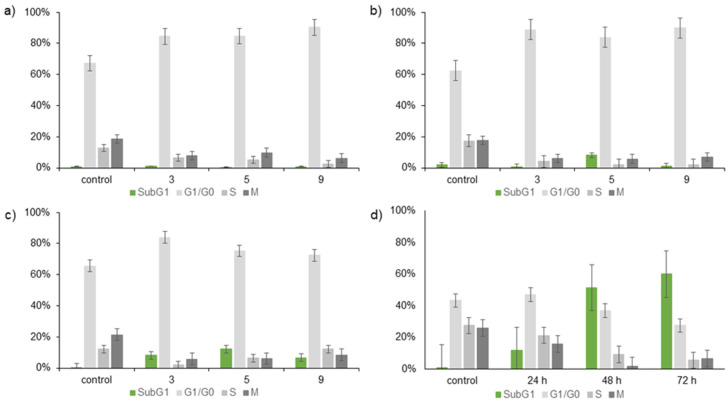
Graphical representation of the cell cycle analysis of the human breast cancer cell line MCF-7 after a treatment period of (**a**) 24 h, (**b**) 48 h, and (**c**) 72 h with compounds **3**, **5**, and **9** and an untreated control. (**d**) Comparison of another cell line (human melanoma; A375) with compound **3** with a treatment period of 24, 48, and 72 h. Double the IC_50_ concentration was used for the treatment.

The MCF-7 breast cancer cell line was used in the experiments. An untreated sample was also included in the cell cycle analysis as a control. The results are depicted in Figure 4.

After 24 h of treatment, all three compounds showed a decrease in S and M phase cells, which can be explained by an increase in G1/G0 phase cells (Figure 4a). Even after 48 h, only compound **5** showed a minimal increase in the number of cells in the SubG1 phase (G1/G0: 83.9%, S: 1.8%, M: 5.8%, SubG1: 8.1%). In addition, after 48 h of treatment, there was a decrease in the S and M phases for all treatments, which is similar to that noted after 24 h. After another 24 h of treatment, the number of cells in the SubG1 phase increased upon treatment with all three substances (**3**: SubG1: 8.1%, **5**: SubG1: 12.1%, **9**: SubG1: 6.5%). As in the 24 and 48 h tests, there was a decrease in M- and S-phase cells and an increase in G1/G0 phase cells after 72 h compared with the untreated control. Several independent experiments by different groups show similar results with MCF-7 [42,43,44]. Due to a lack of DNA fragmentation in MCF-7 cells, the human melanoma cell line A375 was used as a second cell line for comparison (Figure 4d). Since compound **3** showed the most potent effect in the previously shown results of the Annexin V assay, this compound was also used for comparison with the other cell line (A375). In this cell line, a slight increase in the SubG1 phase was observed after 24 h of treatment (G1/G0: 46.9%, S: 21.2%, M: 15.8%, SubG1: 11.9%). The effect of compound **3** is even more pronounced with an incubation time of 48 or 72 h. An increase in the SubG1 peak was observed from 0.7% in the control to up to 60% after 72 h of treatment. These results proved that compound **3** has a strong effect on cell cycle and DNA fragmentation.

### 3.4. Inhibition of Carbonic Anhydrase IX

It has long been known that carbonic anhydrase is capable of cleaving the acetyl group from the artificial substrate *p*-nitrophenyl acetate, resulting in the formation of *p*-nitrophenol (dissociation to *p*-nitrophenolate with an absorbance at λ_max_ = 405 nm) as a product [45,46]. The in vitro assay provides additional information about the potential inhibiting interaction of the conjugates and the target hCA IX. 

Table 3 summarizes the calculated *K_i_* values (competitive inhibition) for the newly synthesized compounds and for AZA and U-104. The latter showed efficient inhibition of hCA IX activity when *p*-nitrophenyl acetate was used as a substrate. The *K_i_* values determined for acetazolamide and U-104 (0.094 µM/0.128 µM) were in the same order of magnitude as reported from the stopped-flow CO_2_ assay commonly used in the literature (*K_i_* 0.025 μM/0.045 µM) [20,47]. Slight deviations can occur from differences in detection method and reaction conditions.

**Figure 5:**
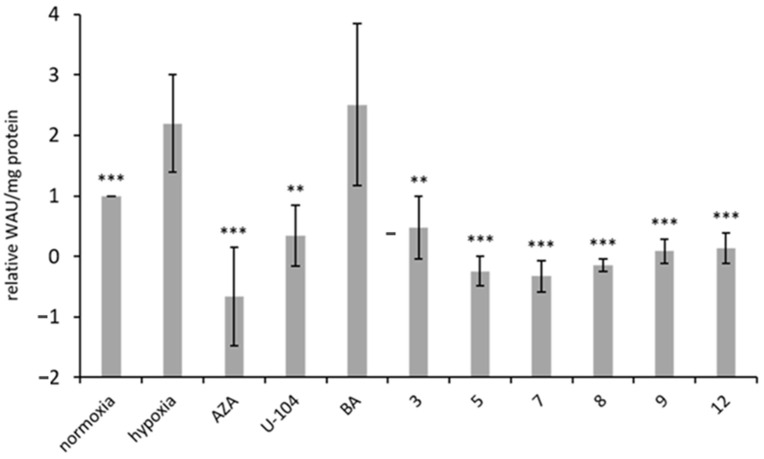
Activity of hypoxia induced hCA IX in a cell-based system measured using the Wilbur–Anderson method. Mean relative Wilbur–Anderson units/mg protein (relative WAU/mg protein) from at least three independent biological replicates are shown. *p* values indicate significant differences from untreated hypoxic cells (** *p* ≤ 0.01; *** *p* ≤ 0.001).

In the present work, acetazolamide with a *K_i_* of 94 nM was used as a reference system for all newly synthesized compounds. It is interesting to note that removal of the acetyl group (compound **6**) only slightly increased the *K_i_* value to 185 nM. The attachment of the linker group (compounds **7** and **8**) had no negative effect on the inhibitory activity against hCA IX, and the *K_i_* values remained in the nanomolar range (**7**: *K_i_* = 129 nM; **8**: *K_i_* = 146 nM). Also, for the combination of the sulfonamide linker adducts with betulin or BA, compounds **3**, **5**, and **9**, we were able to determine inhibitory constants towards hCA IX. The *K_i_* values measured were approximately 10-fold higher than for the unconjugated inhibitors, allowing the conclusion that the betulin/BA moiety of the conjugates impairs proper binding in the active site but still allows the targeting of the enzyme. Due to the bulky structure of the cytotoxin, it can be easily understood that the on- and/or the off-rate that determine *K_i_* are influenced especially if a certain flexibility should be maintained for linker cleavage (see 2.5). For compound **12**, it was not possible to determine a *K_i_* value under the tested conditions with the artificial substrate. Nevertheless, the reduction of activity by 40% with 10 µM of the compound was detectable, concluding that compound **12** is able to interact with the enzyme per se. Our experiments clearly show that the newly synthesized molecules are promising candidates for our purpose using hCA IX as a target to deliver the cytotoxin betulin/BA in near proximity of CAIX-overexpressing tumor cells. In further experiments, we were then interested whether the drug target conjugates show hCA IX inhibition in a cell-based context. Therefore, the Wilbur–Anderson method was used to determine the inhibitory effects of AZA, U-104, BA, and the newly synthesized compounds **3**–**12** against hCA IX. The Wilbur–Anderson method is a cell-based analysis of the change in extracellular pH due to the activity of hCA IX, which is overexpressed under hypoxic conditions. hCA IX activity was calculated using extracellular pH measurement, and the relative Wilbur–Anderson activity (relative WAU) is shown in Figure 5. The inhibitors AZA and U-104 showed a significant reduction in hypoxia-induced hCA IX activity. BA had no potential to inhibit hCA IX activity. In addition, compounds **3–12** (inhibitory fragments and betulin sulfonamides) showed significant inhibition of hypoxia-induced hCA IX activity. Both methods confirm the high potential of the molecules in our drug targeting context. 

### 3.5. Computational Studies

**Figure 6:**
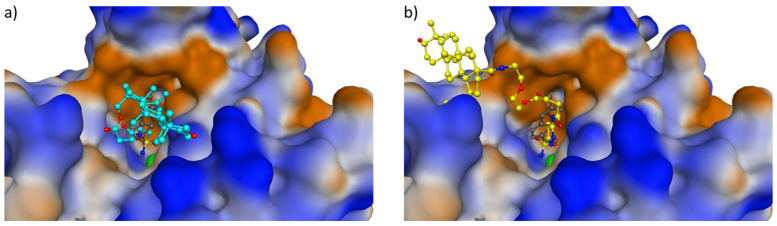
Docking poses of the betulin derivatives **5** (**a**) and **12** (**b**) in the binding pocket of hCA IX superimposed on the crystal structure of AZA (PDB code: 3iai). The surface of the binding pocket was colored by hydrophobicity (blue: hydrophilic, grey: neutral and brown: lipophilic). The zinc(II) ion is represented as a green sphere in the center of the binding pocket.

Due to the large binding pocket of hCA IX, there is sufficient space to accommodate the potential drug molecules proposed in this work. Our intention was that the amide or ester groups in the semi-labile linker would be enzymatically cleaved by proteases or esterases at the site of action, releasing the cytotoxic agent. For this to occur, the cleavage site must be freely accessible to the enzymes and therefore located at the edge or outside of the binding pocket. To get an idea of the positioning of the drug molecules in the binding pocket, the four compounds **3**, **5**, **9**, and **12** were docked into the binding pocket using MOE (The Molecular Operating Environment). Figure 6 shows an example of the obtained binding poses for compounds **5** and **12**. Accordingly, the pentacyclic triterpene backbone of compound **5** (and compound **3**) is located at the edge of the binding pocket, and the bond to be cleaved is located deep inside the pocket. In contrast, the scissile amide bond in compound **12** is much more accessible to enzymes due to the extended linker. The same is true for compound **9**, although the corresponding amide bond is already in the region of the binding pocket.

**Figure 7:**
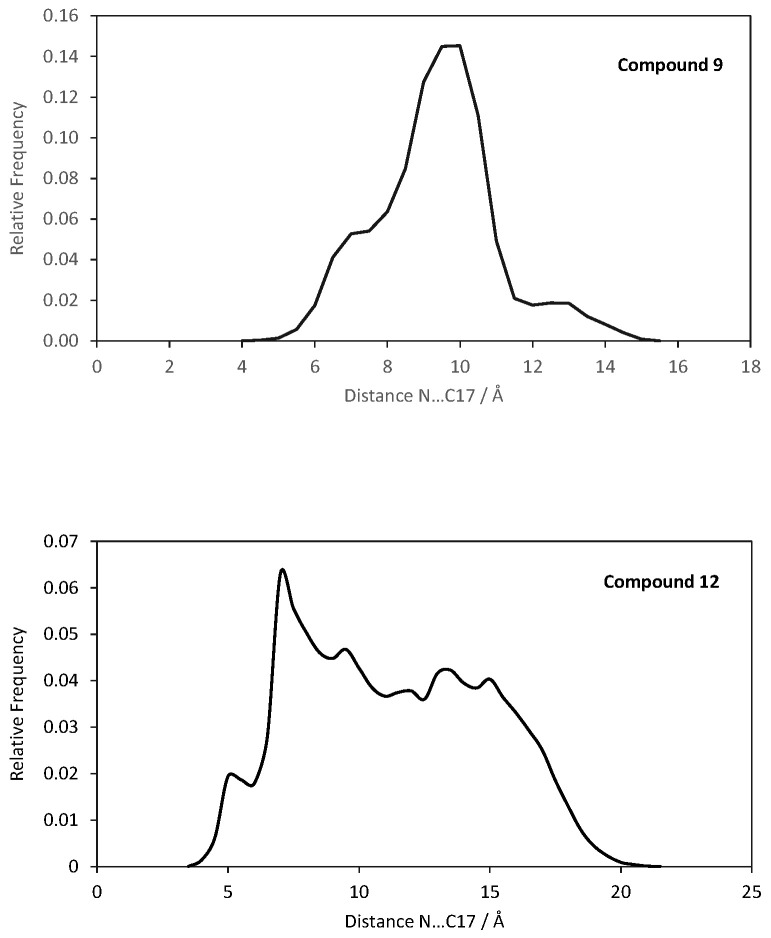
Histograms of distances between the C17 of betulin scaffold and the N atom of the sulfonamide inhibitor for compounds **9** and **12**.

Next, we wanted to know if the extended linkers of compounds **9** and **12** could adopt the stretched conformations necessary for bond cleavage in solution. To this end, molecular dynamics (MD) simulations of compounds **9** and **12** in a water box were performed using the Amber program [48,49]. During the MD simulations, hydrophobic interactions between the heterocyclic ring residue and the betulin scaffold were detected, being enabled by conformational transformations of the flexible bonds of the linker. The histograms for the distance between C17 of the betulin backbone and the nitrogen atom of the sulfonamide group, shown in Figure 7, indicate that the two compounds are predominantly not in a stretched conformation in solution which would be characterized by C17...N distances of approximately 15 and 22 Å. In order to fit into the binding pocket of hCA IX, the more folded conformations of compounds **9** and even more that of **12** have to change to an elongated arrangement. This is an energetically unfavorable process, which is commensurate with the comparatively high *K_i_* values of the two compounds (see Table 3).

The comparison of cytotoxicity (Table 1 and Table 2) and hCA IX inhibition (Table 3 and Figure 5) values of the investigated compounds shows that the CAI component is only of minor importance for cytotoxicity in tumor cells. The inhibitory fragments effectively inhibit hCA IX and show similar *K_i_* values to AZA. However, their cytotoxicity in tumor cells is very low. Conjugates **3**, **5**, and **9** also inhibit hCA IX, but they are 10-fold less active (enzymatically catalyzed hydrolysis of *p*-nitrophenyl acetate) compared to the inhibiting fragments. On the other hand, conjugates **3**, **5**, and **9** have a similarly strong cytotoxic effect as BA and its derivatives. This indicates that changing the structure by adding a CA IX inhibitory fragment via a short linker does not lead to significant changes in cytotoxicity.

However, binding of the hCA IX inhibitor fragments to compound **11** via an extended linker results in compound **12** and leads to a drastic decrease in cytotoxicity and minor inhibition in the enzymatic hydrolysis of *p*-nitrophenyl acetate. It can be assumed that the prolongation of the linker is responsible for those effects. It is clear that our structure-property analysis is not yet possible on the basis of these results. Further computational studies will be essential to make progress.

Measurement of hCA IX activity in a cell-based system using extracellular pH measurement shows significant inhibition of hypoxia-induced hCA IX activity for all tested compounds **3**–**12** (inhibitory fragments and betulin sulfonamides) and confirm the high potential of the molecules in our drug targeting context. 

In summary, we have described the synthesis, biological investigation and inhibitory effects of new betulin and BA derivatives (**3**, **5**, **9**, and **12**) on hCA IX and also computational studies that estimate the interaction with the enzyme and their three-dimensional arrangement. The compounds were designed in such a way that an inhibitory component was bound to a cytotoxic agent (betulin/BA fragment) via a linker. The underlying idea was to develop a conjugate that offers the possibility of accumulating a cytotoxic molecule in the tumor tissue using hCA IX as a target structure. Betulin and BA were chosen as the cytotoxic part, and 2-aminoethane-1-sulfonamide and 5-amino-1,3,4-thiadiazole-2-sulfonamide were selected as the hCA IX-inhibiting part. The linker should be cleaved so that the cytotoxic part of the molecule can enter the tumor cell, whereas the hCA IX-inhibiting part remains in the enzyme. Further studies are needed to test these therapeutic strategies using in vivo models. This is particularly important as hypoxia-induced hCA IX promotes tumor cell survival and acidification of the microenvironment, resulting in increased tumor malignancy, invasiveness, and resistance to therapy.

## 4. Conclusions

Overall, with the synthesis of conjugates of betulin/BA, a linker, and CA IX inhibitor fragments, the dual strategy to produce effective therapeutics that can inhibit tumor-associated hCA IX has been successfully implemented in in vitro tumor models. However, this concept still needs to be validated in in vivo models and in clinical applications, as the targeted use of drugs against therapy-resistant hypoxic tumor cells could be an important milestone in improving the tumor therapy in general.

## Figures and Tables

**Table 1:** Cytotoxicity of the sulfonamides and acetazolamide conjugates shown by IC_50_ values [µM] from SRB assays after an incubation period of 96 h in three cancer cell lines (A375—melanoma, MCF-7—breast cancer and A2780—ovarian cancer). Data are the mean (±SD) of at least three independent experiments, each performed in triplicate.

Compound	A375	MCF-7	A2780
**AZA**	>100	>100	>100
**BA**	13.1 ± 0.9	13.0 ± 1.0	12.5 ± 1.8
**3**	6.4 ± 0.7	7.5 ± 0.7	10.4 ± 0.8
**4**	12.1 ± 1.5	9.8 ± 0.6	12.3 ± 3.9
**5**	9.4 ± 0.5	8.6 ± 0.7	11.4 ± 1.4
**6**	>100	>100	>100
**7**	>100	>100	>100
**8**	>100	>100	>100
**9**	7.2 ± 0.6	6.2 ± 0.2	8.7 ± 2.0
**10**	4.3 ± 0.2	5.9 ± 0.2	9.5 ± 0.9
**11**	10.5 ± 0.6	7.3 ± 0.4	12.3 ± 1.5
**12**	>100	>100	35.1 ± 3.4

**Table 2:** Spheroid cytotoxicity assay: IC_50_ values [µM] after 7 days of treatment averaged from 3 independent experiments, comprising 8 spheroids per concentration; human breast cancer cell line-derived spheroids: MDA-MB-231, Hs578T.

Compound	MDA-MB-231	Hs578T
**U-104**	33.5 ± 7.6	83.9 ± 12.4
**5**	10.3 ± 2.7	30.1 ± 0.7
**7**	>100	>100
**8**	>100	>100
**9**	8.1 ± 1.4	28.4 ± 0.3
**12**	>100	>100

**Table 3:** Inhibition data for the sulfonamides investigated in the present study and standard CA inhibitors against hCA IX; n.i., no inhibition; r.a., residual activity.

Compound	*K_i_* Values [µM] of hCA IX
**AZA**	0.094 ± 0.027
**U-104**	0.128 ± 0.045
**BA**	n.i.
**3**	1.04 ± 0.39
**5**	1.1 ± 0.31
**6**	0.185 ± 0.04
**7**	0.129 ± 0.05
**8**	0.146 ± 0.05
**9**	1.25 ± 0.25
**12**	r.a. (10 µM): 60%

## Data Availability

Data are contained within the article.

## Supplementary Materials

The following supporting information can be downloaded at: https://www.mdpi.com/article/10.3390/pharmaceutics16030401/s1, Figure S1: Representative ^1^H- and ^13^C-NMR spectra and ESI-MS spectra of the compounds synthesized in this work.
