# Supplementary material for: Combination of Betulinic Acid Fragments and Carbonic Anhydrase Inhibitors—A New Drug Targeting Approach
Source: Pharmaceutics. 2024 Mar 14;16(3):401. doi: 10.3390/pharmaceutics16030401 (PMC10974084; doi:10.3390/pharmaceutics16030401)
Supplement: Supplementary file 1 [file pharmaceutics-16-00401-s001.zip › pharmaceutics-2887787-supplementary.pdf]

Supplementary Materials

Combination of Betulinic Acid Fragments and Carbonic Anhydrase Inhibitors – A New Drug Targeting Approach

Matthias Bache [1,*], Niels V. Heise [2], Andreas Thiel [3], Anne Funtan [4], Franziska Seifert [4], Marina Petrenko [1],
Antje Güttler [1], Sarah Brandt [5], Thomas Mueller [5], Dirk Vordermark [1], Iris Thondorf [3], René Csuk [2] and Reinhard Paschke [4]

[1] Department of Radiotherapy, Martin-Luther-University Halle-Wittenberg, Halle (Saale),
[2] Institute of Chemistry, Martin-Luther-University Halle-Wittenberg, Halle (Saale).
[3] Institute of Biochemistry and Biotechnology, Martin-Luther-University Halle-Wittenberg, Halle (Saale),
[4] BioCenter, Martin-Luther-University Halle-Wittenberg, Halle (Saale),
[5] Department of Internal Medicine IV (Hematology/Oncology), Martin-Luther-University Halle-Wittenberg, Halle (Saale).
* Correspondence: matthias.bache@uk-halle.de; Tel.: +495557424

Figure S1: *Representative ^{1}H- and ^{13}C- NMR spectra and ESI-MS spectra of the compounds synthesized in this work*

Compound **3**

^{1}H NMR ($CDCl_3$, 400 MHz)

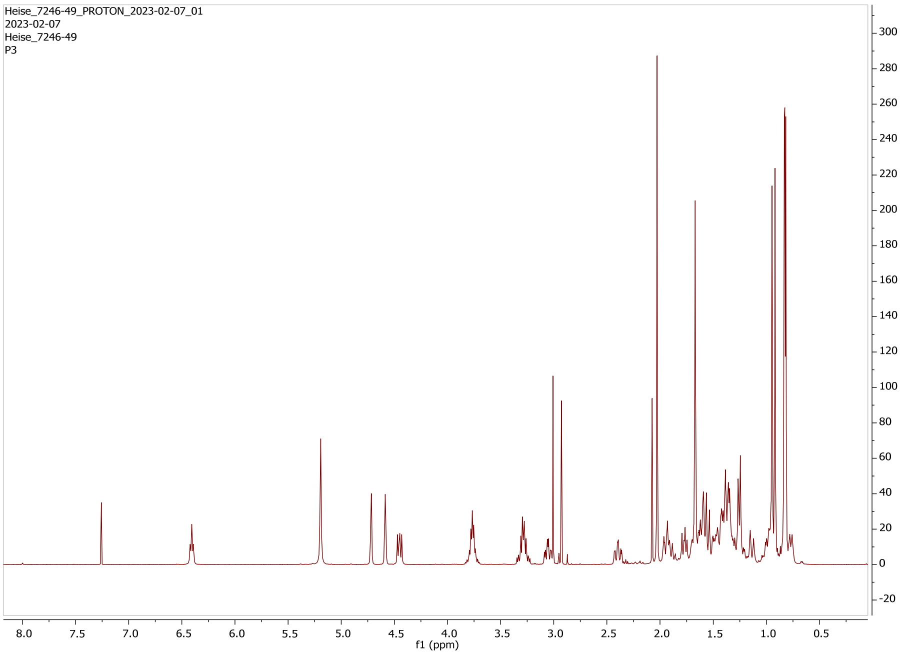

^{13}C NMR ($CDCl_3$, 101 MHz)

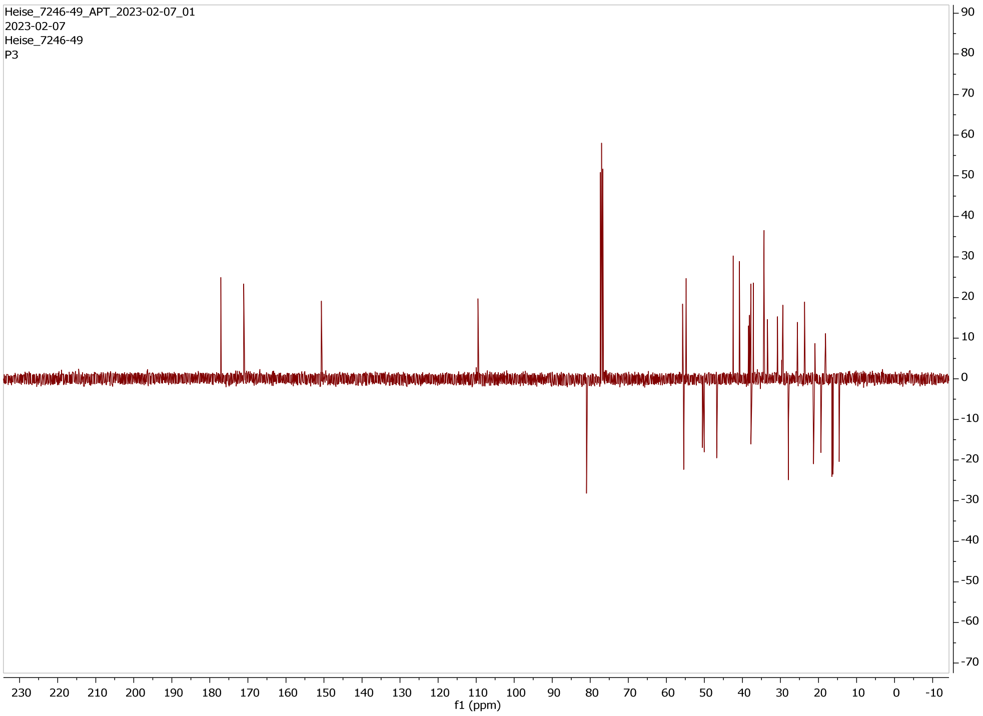

Compound **4**

^{1}H NMR ($CDCl_3$, 500 MHz)

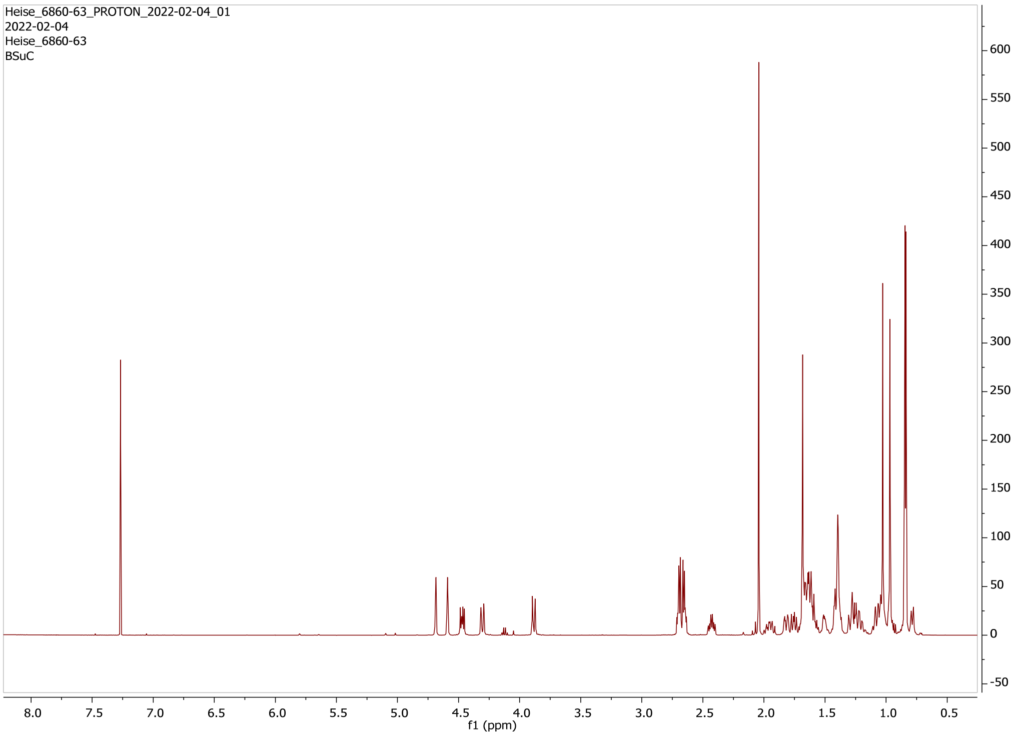

^{13}C NMR ($CDCl_3$, 126 MHz)

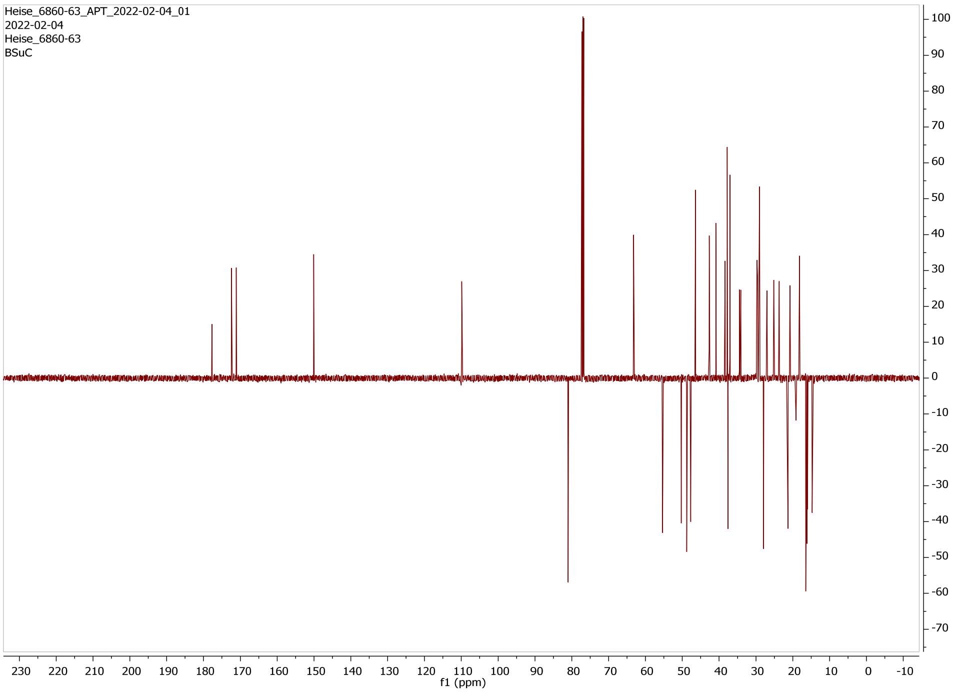

ESI-MS

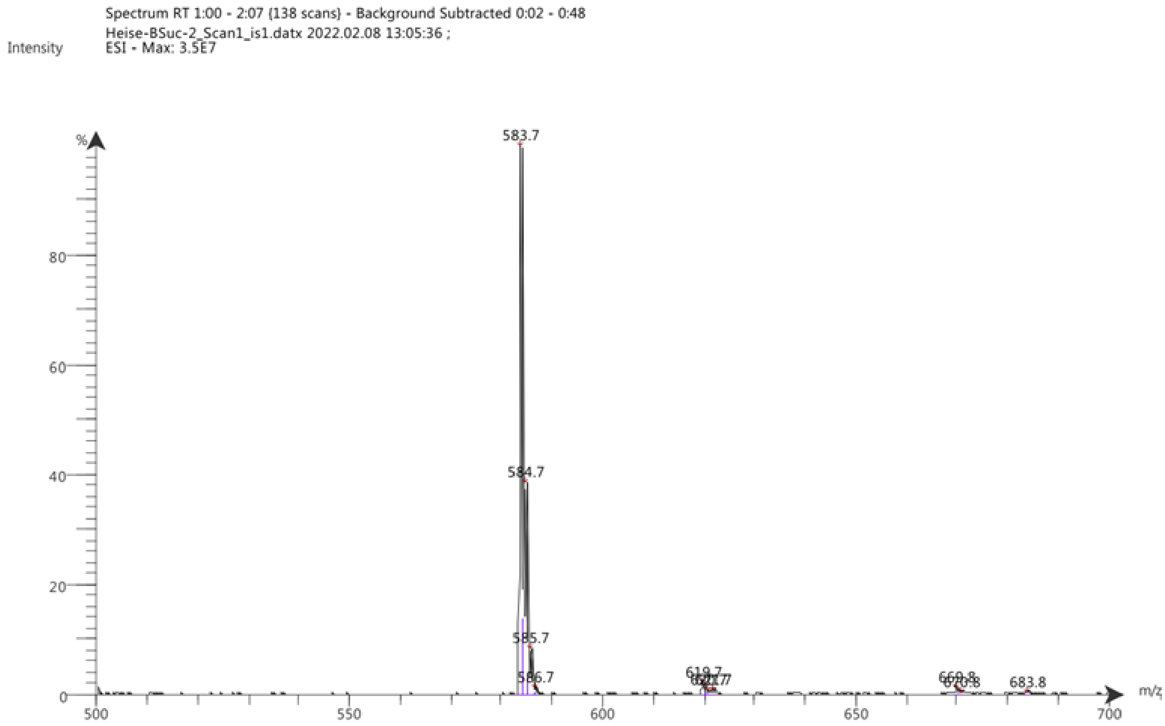

Spectrum RT 1:00 - 2:07 (138 scans) - Background Subtracted 0:02 - 0:48
Heise-BSuc-2_Scan1_is1.datx 2022.02.08 13:05:36 ;
ESI - Max: 3.5E7
Intensity
%
583.7
584.7
585.7
586.7
619.7
683.8
m/z

Compound **5**

^{1}H NMR ($CDCl_3$, 500 MHz)

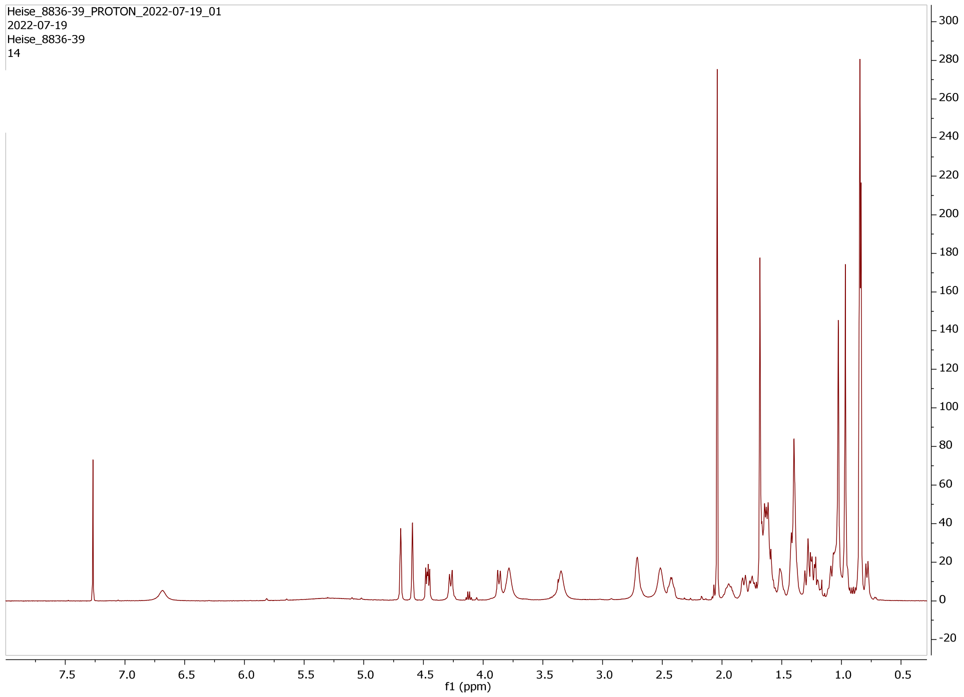

^{13}C NMR ($CDCl_3$, 126 MHz)

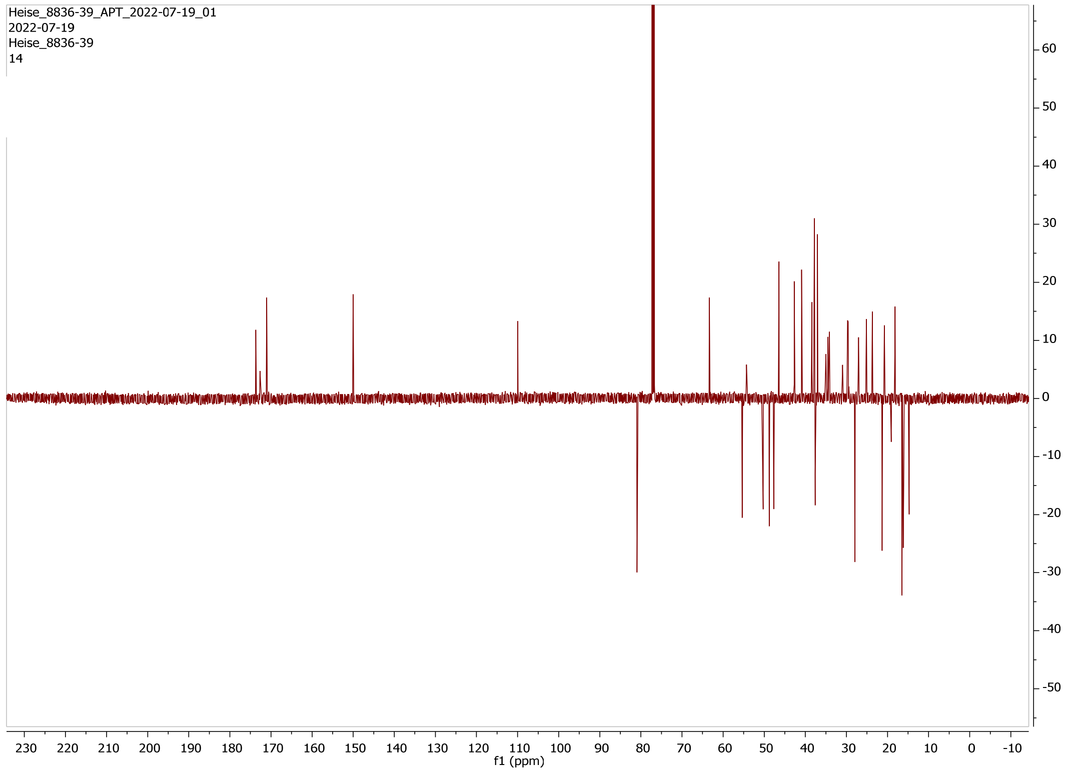

ESI-MS

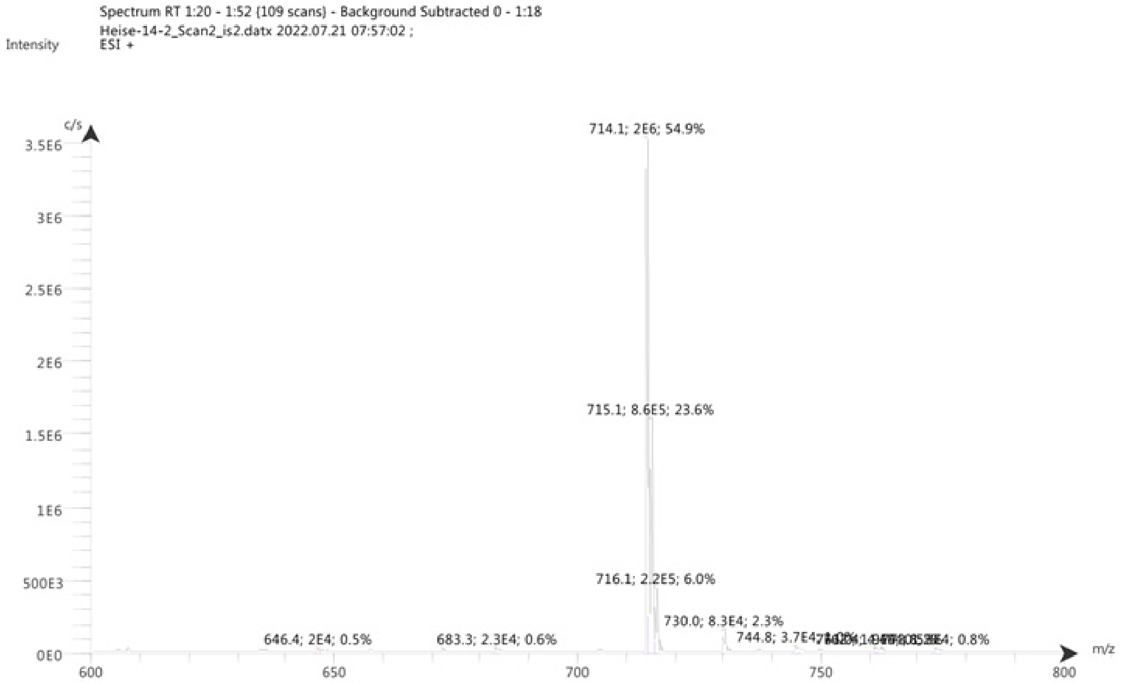

Spectrum RT 1:20 - 1:52 (109 scans) - Background Subtracted 0 - 1:18
Heise-14-2_Scan2_is2.datx 2022.07.21 07:57:02 ;
ESI +
Intensity
c/s
714.1; 2E6; 54.9%
715.1; 8.6E5; 23.6%
716.1; 2.2E5; 6.0%
730.0; 8.3E4; 2.3%
744.8; 3.7E4
646.4; 2E4; 0.5%
683.3; 2.3E4; 0.6%
m/z

Compound **6**

^{1}H NMR (DMSO-d_6, 500 MHz)

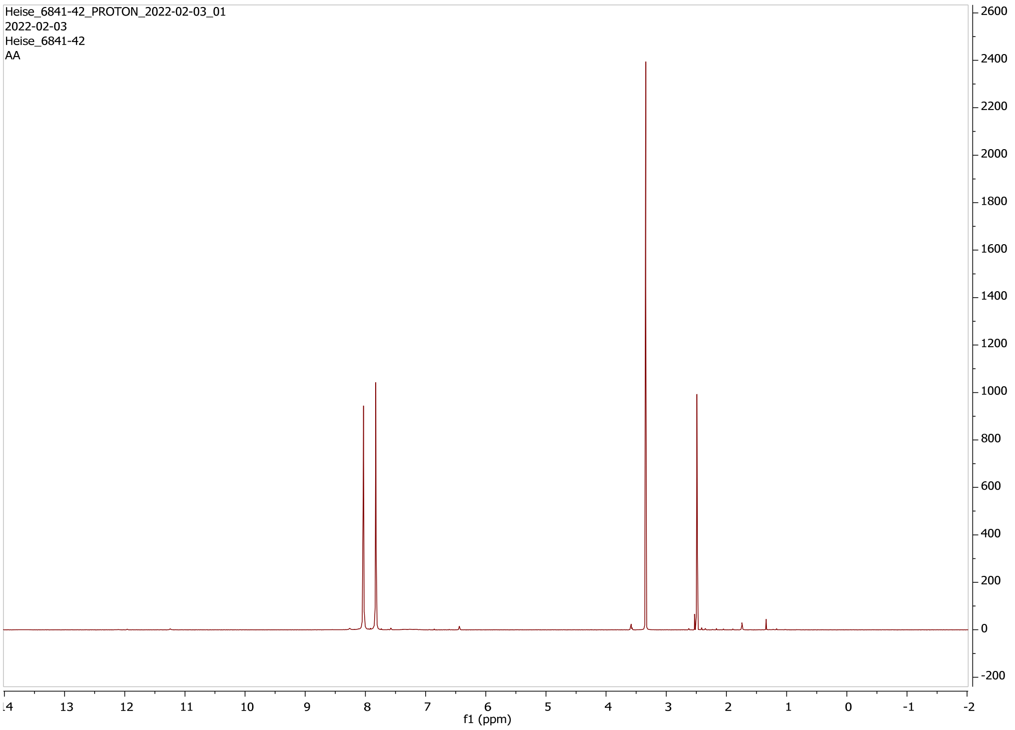

^{13}C NMR (DMSO-d_6, 126 MHz)

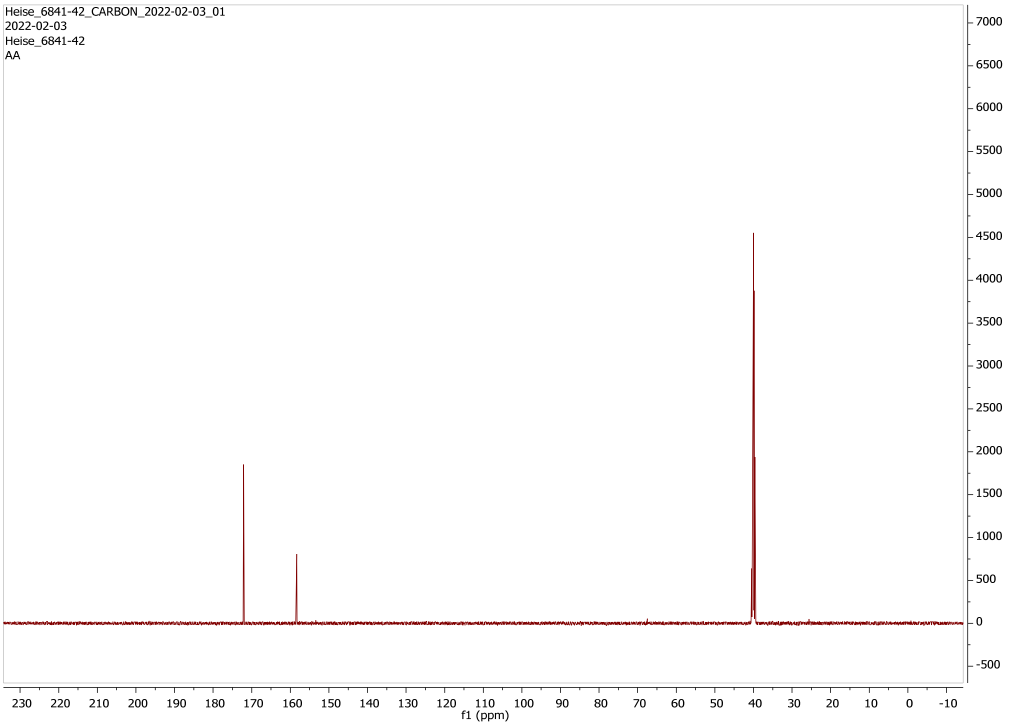

ESI-MS

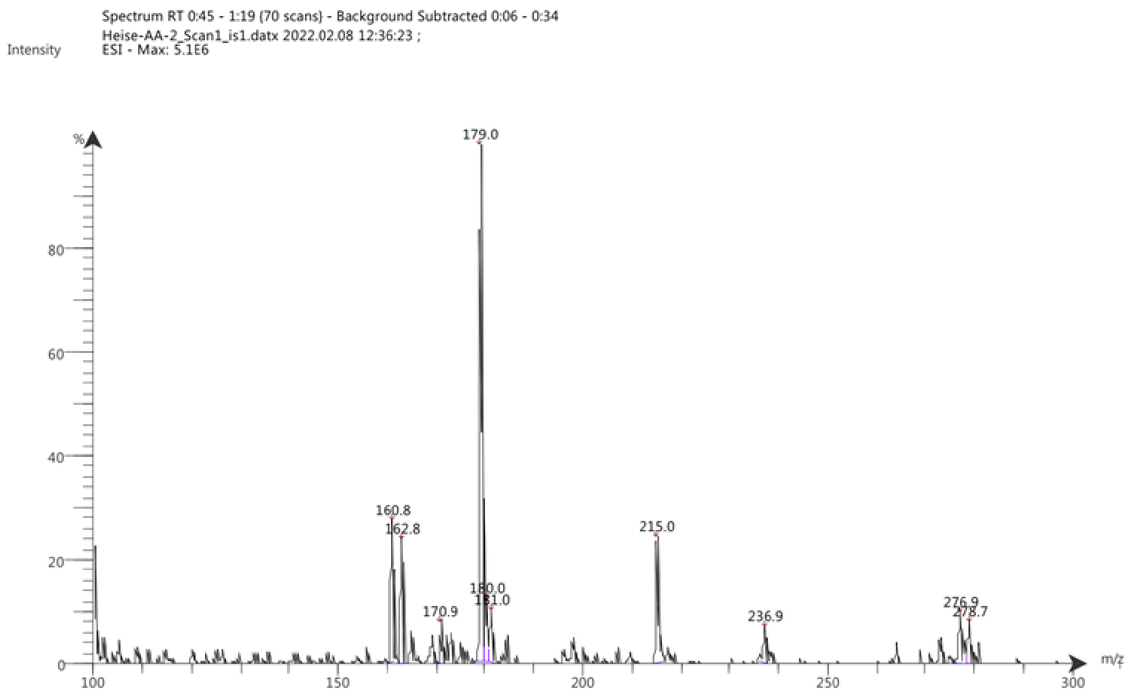
Spectrum RT 0:45 - 1:19 (70 scans) - Background Subtracted 0:06 - 0:34
Heise-AA-2_Scan1_is1.datx 2022.02.08 12:36:23 ;
ESI - Max: 5.1E6
Intensity
%
179.0
160.8
162.8
215.0
180.0
181.0
170.9
236.9
276.9
278.7
m/z
0
20
40
60
80
100
150
200
250
300

Compound **9**

^{1}H NMR (DMSO-d$_6$, 400 MHz)

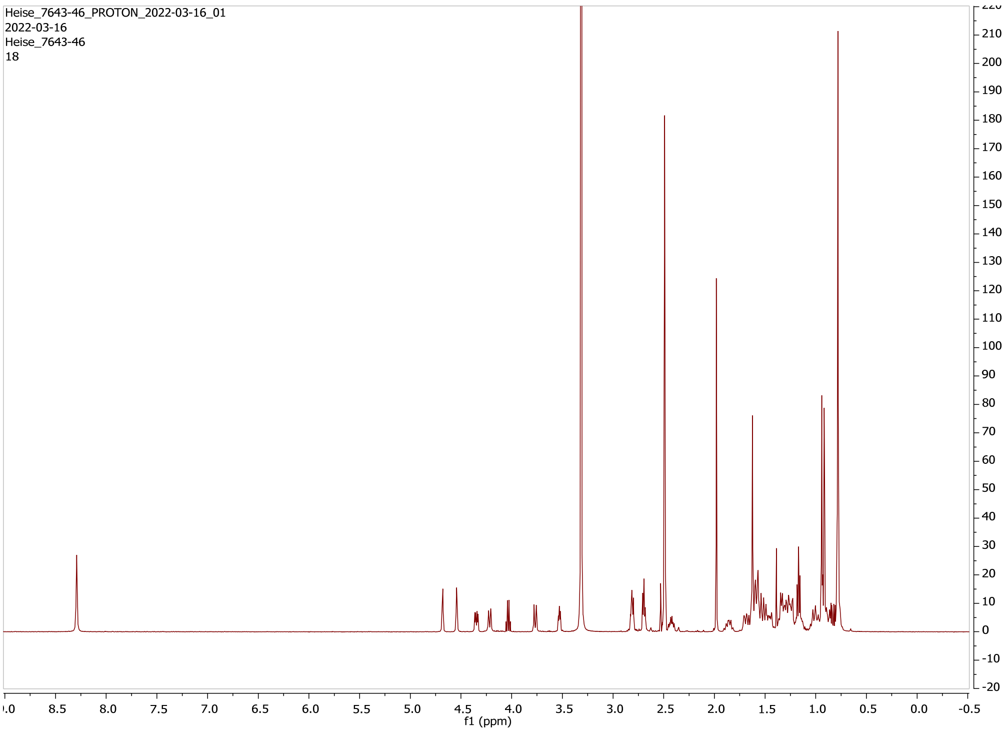

^{13}C NMR (DMSO-d$_6$, 101 MHz)

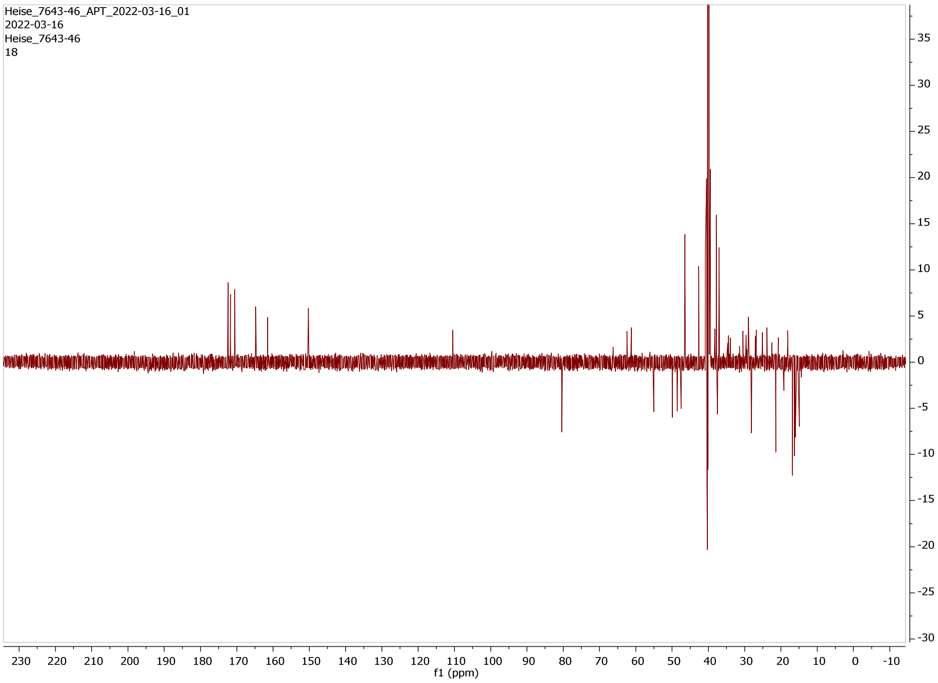

ESI-MS

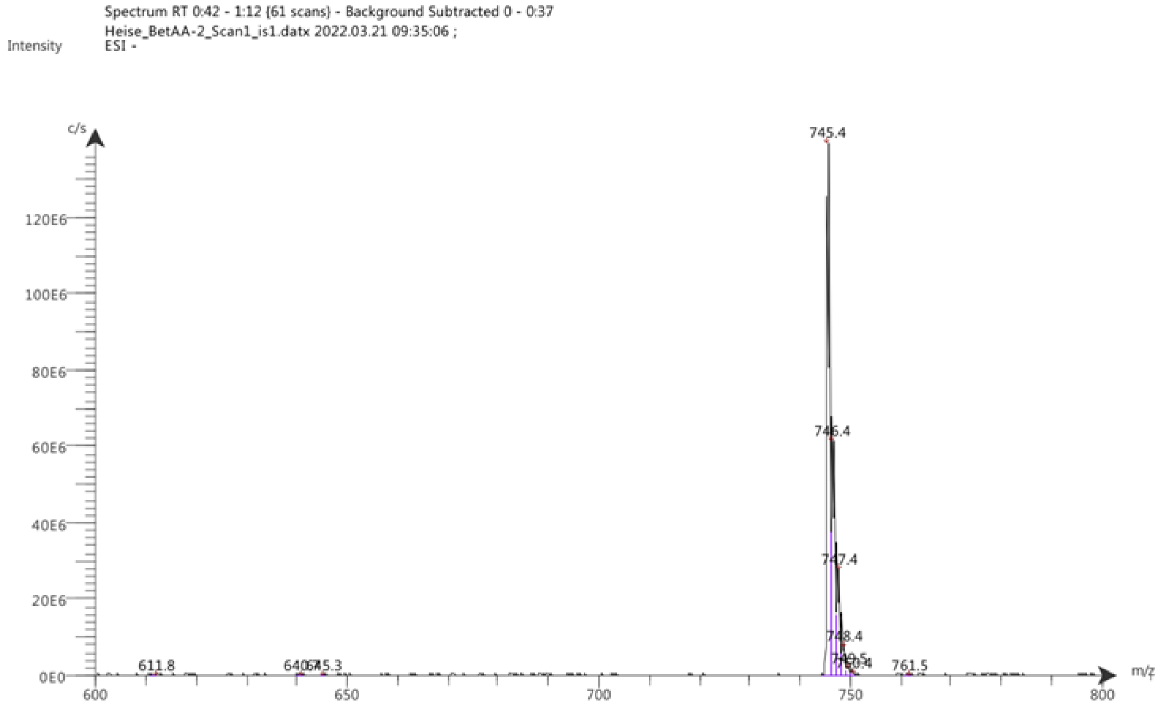
Spectrum RT 0:42 - 1:12 (61 scans) - Background Subtracted 0 - 0:37
Heise_BetAA-2_Scan1_is1.datx 2022.03.21 09:35:06 ;
ESI -
Intensity
c/s
120E6
100E6
80E6
60E6
40E6
20E6
0E0
745.4
746.4
747.4
748.4
611.8
761.5
600
650
700
750
800
m/z

Compound **10**

^{1}H NMR ($CDCl_3$, 400 MHz)

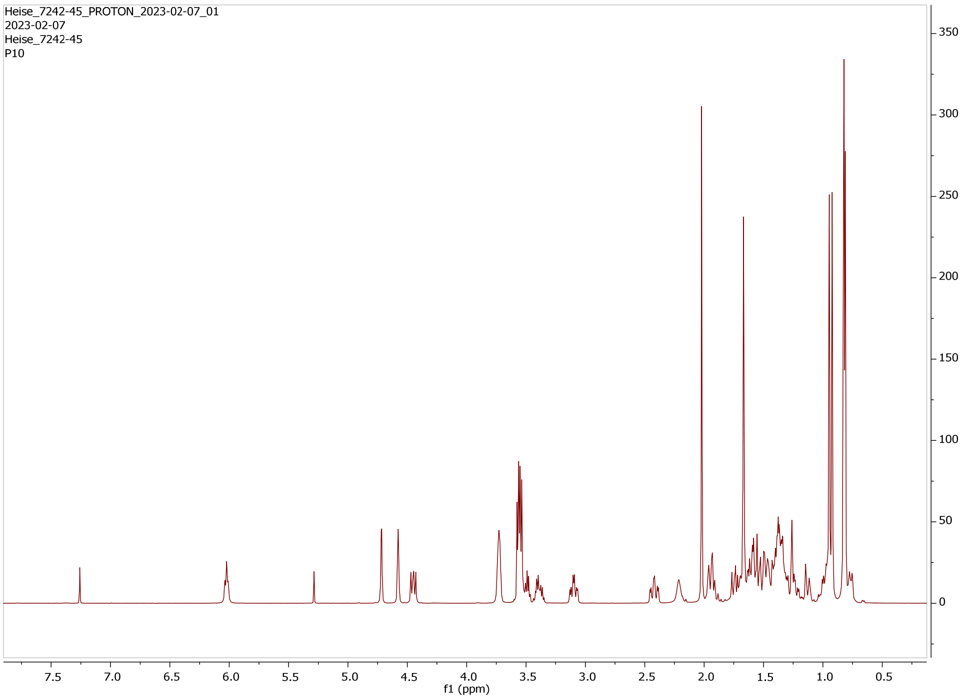

^{13}C NMR ($CDCl_3$, 101 MHz)

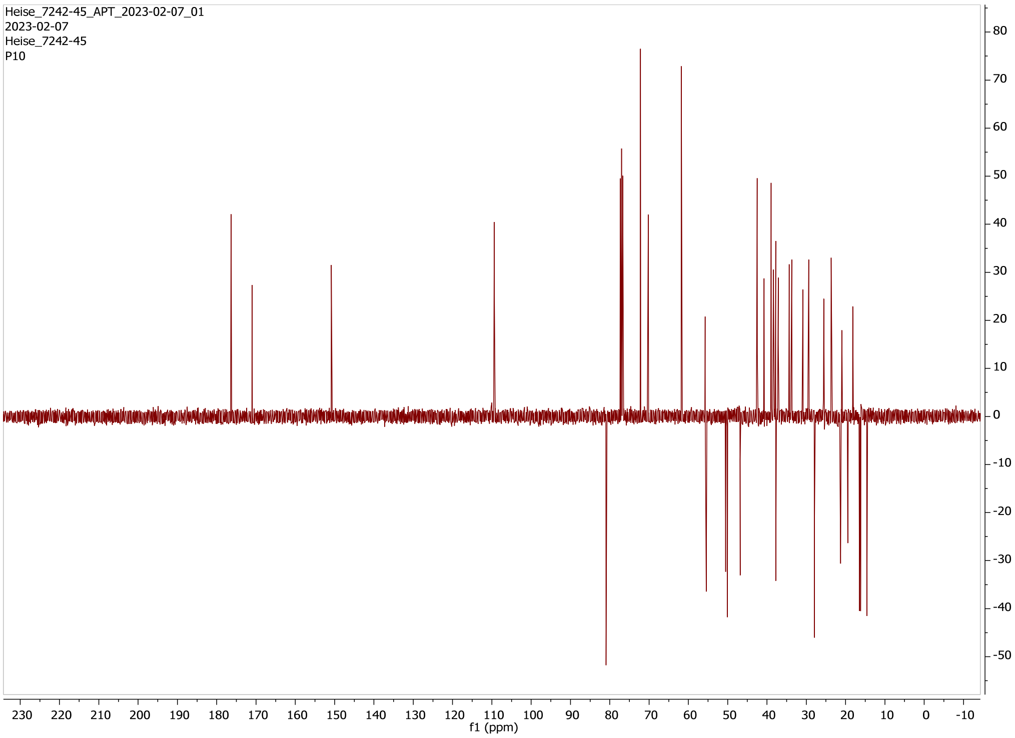

Compound **11**

^{1}H NMR ($CDCl_3$, 400 MHz)

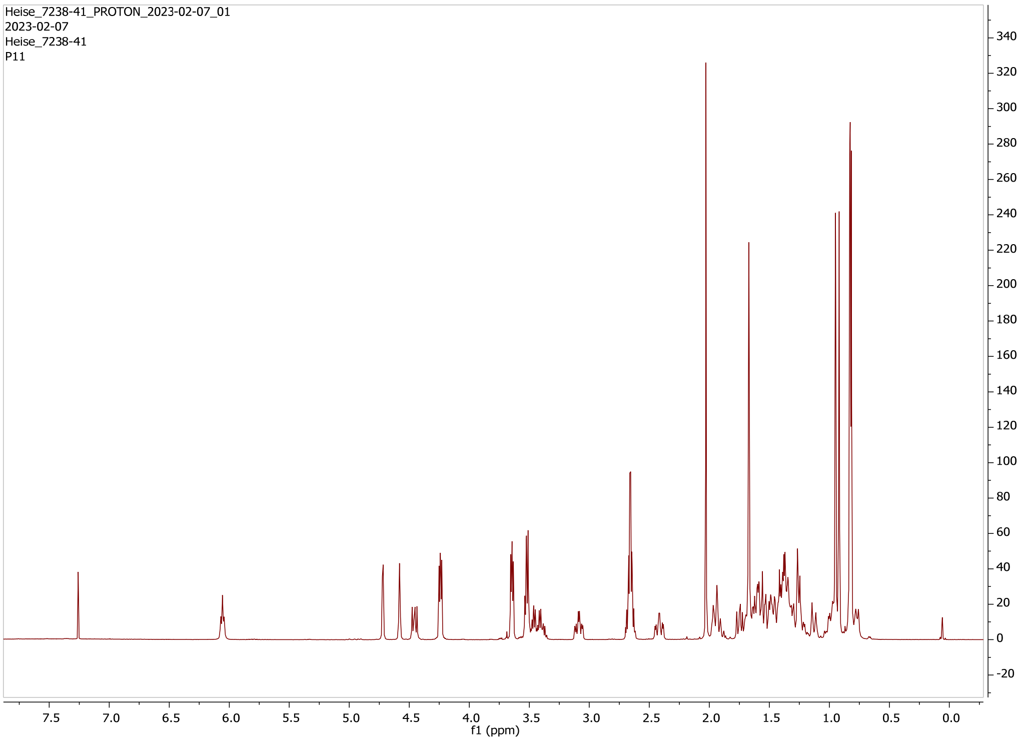

^{13}C NMR ($CDCl_3$, 101 MHz)

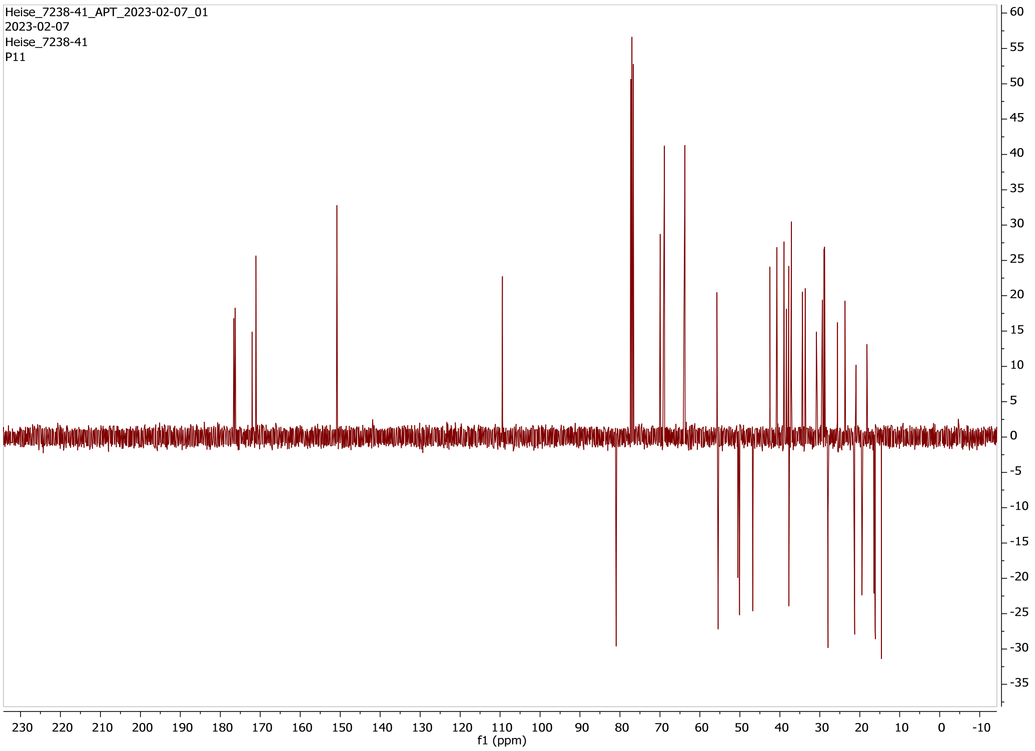

Compound **12**

^{1}H NMR ($CDCl_3$, 500 MHz)

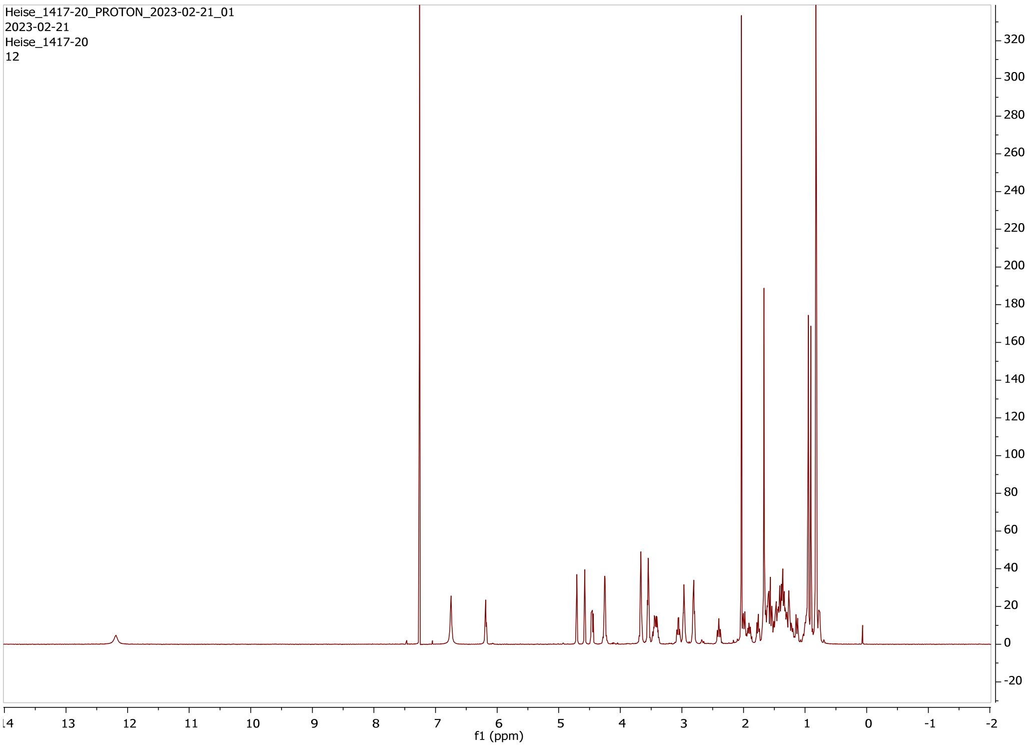

^{13}C NMR ($CDCl_3$, 126 MHz)

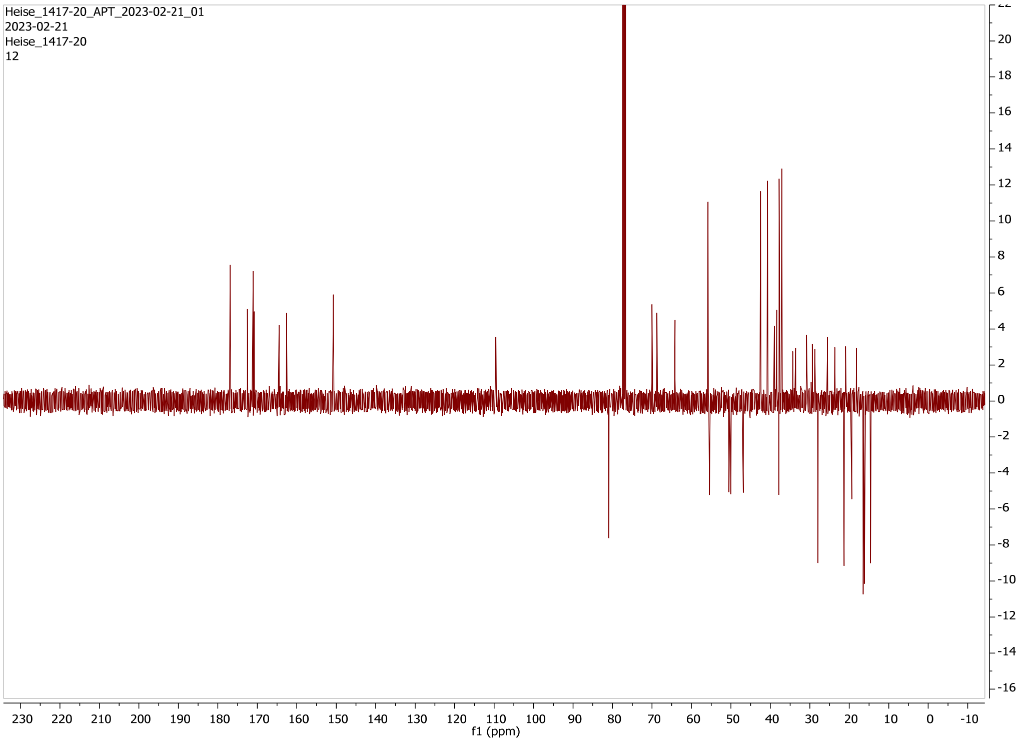

ESI-MS

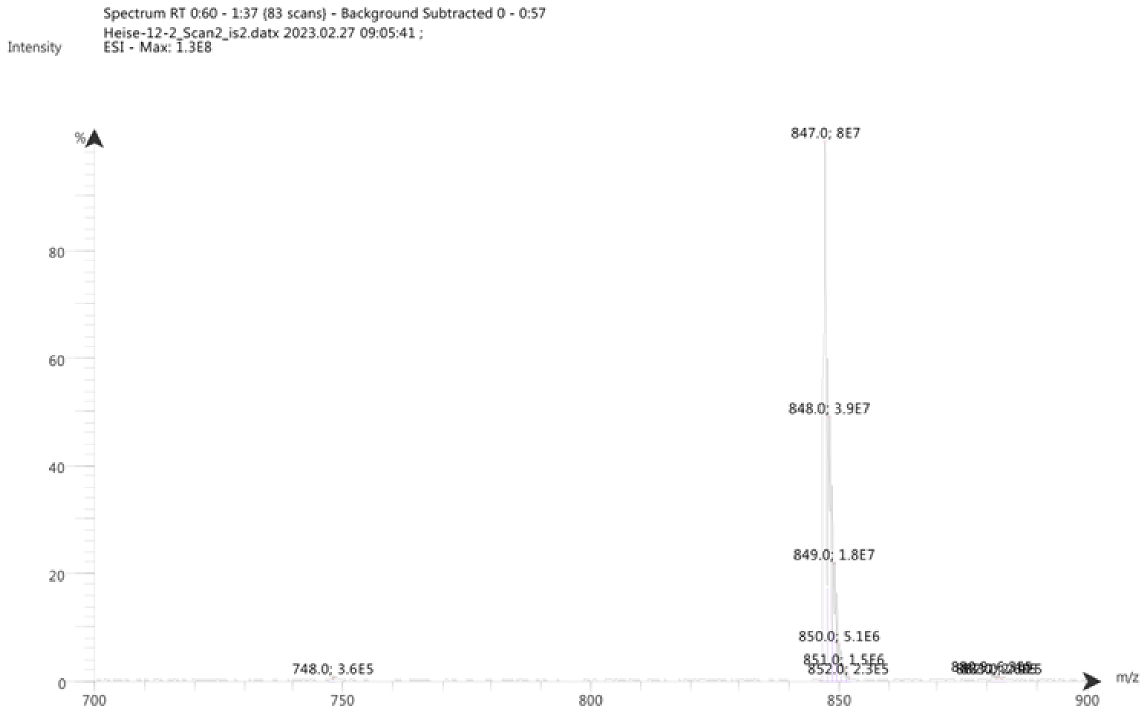
Spectrum RT 0:60 - 1:37 (83 scans) - Background Subtracted 0 - 0:57
Heise-12-2_Scan2_is2.datx 2023.02.27 09:05:41 ;
ESI - Max: 1.3E8
Intensity
%
847.0; 8E7
848.0; 3.9E7
849.0; 1.8E7
850.0; 5.1E6
851.0; 1.5E6
852.0; 2.3E5
748.0; 3.6E5
m/z
0
20
40
60
80
700
750
800
850
900
